# Similar but Not Identical—Binding Properties of LSU (Response to Low Sulfur) Proteins From *Arabidopsis thaliana*

**DOI:** 10.3389/fpls.2020.01246

**Published:** 2020-08-14

**Authors:** Anna Niemiro, Dominik Cysewski, Jerzy Brzywczy, Anna Wawrzyńska, Marzena Sieńko, Jarosław Poznański, Agnieszka Sirko

**Affiliations:** ^1^Department of Plant Biochemistry, Institute of Biochemistry and Biophysics Polish Academy of Sciences, Warsaw, Poland; ^2^Laboratory of Mass Spectrometry, Institute of Biochemistry and Biophysics Polish Academy of Sciences, Warsaw, Poland; ^3^Department of Biophysics, Institute of Biochemistry and Biophysics Polish Academy of Sciences, Warsaw, Poland

**Keywords:** hub proteins, protein–protein interaction, coiled-coil, plant stress response, sulfur starvation, yeast two hybrid approach, Bimolecular Fluorescent Complementation

## Abstract

Members of the plant-specific LSU (RESPONSE TO LOW SULFUR) family are strongly induced during sulfur starvation. The molecular functions of these proteins are unknown; however, they were identified as important stress-related hubs in several studies. In *Arabidopsis thaliana*, there are four members of the LSU family (LSU1–4). These proteins are small (approximately 100 amino acids), with coiled-coil structures. In this work, we investigated interactions between different monomers of LSU1–4. Differences in homo- and heterodimer formation were observed. Our structural models of LSU1–4 homo- and heterodimers were in agreement with our experimental observations and may help understand their binding properties. LSU proteins are involved in multiple protein–protein interactions, with the literature suggesting they can integrate abiotic and biotic stress responses. Previously, LSU partners were identified using the yeast two hybrid approach, therefore we sought to determine proteins co-purifying with LSU family members using protein extracts isolated from plants ectopically expressing TAP-tagged LSU1–4 constructs. These experiments revealed 46 new candidates for LSU partners. We tested four of them (and two other proteins, CAT2 and NBR1) for interaction with LSU1–4 by other methods. Binding of all six proteins with LSU1–4 was confirmed by Bimolecular Fluorescence Complementation, while only three of them were interacting with LSUs in yeast-two-hybrid. Additionally, we conducted network analysis of LSU interactome and revealed novel clues for the possible cellular function of these proteins.

## Introduction

The *A. thaliana* genome encodes four LSU proteins, LSU1 (At3g49580), LSU2 (At5g24660), LSU3 (At3g49570), and LSU4 (At5g24655). The function of these plant-specific proteins are not well known (Sirko et al., 2015). The genes are located on chromosome 3 and chromosome 5, in two direct repeats of intron-less genes, split by approximately 2.5 kb each. The expression of *LSU* genes are up-regulated by sulfur starvation (−S), not only in *Arabidopsis* (Maruyama-Nakashita et al., 2006) but also in tobacco plants (Lewandowska et al., 2010). Also, the regulation of *LSU* genes by other stresses has been reported (Davletova et al., 2005; Usadel et al., 2008; Ruckle et al., 2012; Garcia-Molina et al., 2017). Moreover, *LSU1* belongs to the so called “O-acetylserine (OAS) gene cluster”, a set of six genes whose transcript levels increase not only during sulfur deficiency, but also with endogenous increases in OAS during normal sulfate nutrition (Hubberten et al., 2012).

LSU proteins are involved in numerous protein–protein interactions with proteins of various molecular functions at different cellular locations. LSU1, LSU2, and LSU3 were identified as immune-related hubs playing undefined roles in response to pathogens, with partially overlapping and relatively large interactomes (Arabidopsis Interactome Mapping Consortium, 2011; Mukhtar et al., 2011; Wessling et al., 2014). LSU4 was not included in these studies, but it was previously suggested to be involved in flower development (Myakushina et al., 2009). Reduced expression of *LSU1/2* causes a moderately enhanced disease susceptibility in plants exposed to abiotic stresses, such as nutrient deficiency, high salinity, or heavy metal toxicity, whereas LSU1 overexpression confers significant disease resistance in several conditions in *Arabidopsis* (Garcia-Molina et al., 2017). Similar effects, as well as changes in transcriptome levels in plants with down-regulated expression of the LSU1–4 homolog (UP9C), were observed in tobacco plants (Lewandowska et al., 2010; Moniuszko et al., 2013).

This protein family is now recognized as a stress-related hub that integrates abiotic and biotic stress responses (Vandereyken et al., 2018). Besides some high-throughput studies suggesting LSU function as immune-related hubs (Arabidopsis Interactome Mapping Consortium, 2011; Mukhtar et al., 2011), several reports describe other partners, including MYB51 as partner to LSU3 (Frerigmann et al., 2014), iron (Fe)-dependent superoxide dismutase (SOD) FSD2 as partner to LSU1 (Garcia-Molina et al., 2017), and 1-aminocyclopropane-1-carboxylic acid (ACC) oxidase (Moniuszko et al., 2013), and selective autophagy cargo receptor Joka2/NtNBR1 as partner to UP9C (a homolog of LSU1–4) in tobacco plants (Zientara-Rytter et al., 2011). The LSU1, LSU2, and LSU3 interactomes, identified by the yeast two hybrid approach (Y2H), show extensive overlaps, but they are not identical (Arabidopsis Interactome Mapping Consortium, 2011; Mukhtar et al., 2011), and it has yet to be ascertained if partners for one LSU can interact with all members of the LSU family.

The amino acid sequences of these proteins (approximately 100 amino acids) are poorly conserved among family members from different plants, except for a short motif (A-x-x-x-E-E-x-L-C-x-x-L-x-[E/D]-x-[E/D]); however, all conformations are predicted to exist in the *α*-helical formation and partially coiled-coil forms (Sirko et al., 2015). Indeed, circular dichroism analysis of recombinant UP9C revealed it was almost entirely *α*-helical (Lewandowska et al., 2010). The 3D structures of LSU-like proteins have not been determined. Despite the fact that family proteins have coiled-coil structures, a feature known to facilitate oligomerization (Burkhard et al., 2001), multimer (homo- and hetero-) formation by these proteins has not yet been considered and not fully examined.

In this work we focus on protein–protein interactions of LSU proteins. We tested the ability of LSU family members to form homo- and heterodimers. To understand and visualize differences in dimer formation, structural 3D models of LSU dimers were created. These models suggest that dimer formation by LSU may serve regulatory purposes and that the dimers bind other molecular partners, rather than monomeric forms do. The proposed structural model was tested by targeted mutagenesis of LSU1, and the effects of particular replacements on binding properties of LSU1 were inspected. Besides, the new candidates for LSU partners were searched using Tandem Affinity Purification-mass spectrometry analyses (TAP-MS), and a few novel direct interactors were endorsed by other methods. We have also analyzed the LSU’s interaction network expecting to gain more info about their possible function.

## Materials and Methods

### Gene Cloning, Vectors and Plasmid Construction

The coding regions of *A. thaliana LSU* genes were amplified from cDNAs using the primers indicated in **Table 1** and re-cloned into Gateway™ vectors using PCR fragment + pENTR/D-TOPO to make an Entry Clone and LR reaction (Entry Clone + pDEST22 or pDEST32 + LR Clonase II to make an Expression Clone) according to the manufacturer’s protocols (Invitrogen, USA). The series of Y2H plasmids encoding the LSU1 protein with C^54^A, C^54^E, C^54^R, and L^60^A mutations were constructed in both Y2H vectors (pDEST22 and pDEST32) by using the respective plasmids containing the wild type *LSU1* gene as a template for the sequential rounds of PCR. For each vector, two types of overlapping PCR products (A and B) were generated using the Phusion High Fidelity DNA polymerase (Thermo Fisher Scientific, Boston, MA, USA) and the following pairs of primers. For the type A products: the GAL4-AD or GAL4-BD primers (specific for the sequences in the pDEST22 or pDEST32 vector, respectively) and reverse mutagenic primers converting the cysteine codon TGC to an alanine (GCA) or a glutamine (GAG), or an arginine (CGA) codon and in the case of leucine mutagenesis converting CTG codon to an alanine (GCA) one, were used. For the type B products: the forward mutagenic primers and the reverse tADH1R primer (specific for sequences in the pDEST22 and pDEST32 vectors) were used. Both types of the products (A and B) were purified from an agarose gel and used as templates for the next rounds of the corresponding PCR reactions with the vector specific primers. The final products containing the full-length mutated genes were digested with *Nco*I and *Pau*I (Thermo Fisher Scientific, Boston, MA, USA), and the restriction fragments were transferred to the corresponding plasmid containing the wild type *LSU1* digested by the same enzymes exchanging original sequence to the sequence with mutation. All clones were validated by sequencing.

**Table 1:** Oligonucleotides used as primers.

AGI	Gene symbol or primer name	Oligos (5′–3′) used for PCR or mutagenesis (*LSU1^C54^ LSU1^L60^*); F—forward, R—reversed
At3g49580	*LSU1*	F: CACCATGGCGAACCGAGGAGGAT R: CGAGGAAGAGACGACAGAAGAAG
At5g24660	*LSU2*	F: CACCATGGGGAAAGGAGGAAAC R: CGGAGAGGCAGAGGCAGA
At3g49570	*LSU3*	F: CACCATGGGAAAAGGAGGAGGT R: CGAATTCGTAACAACGAC
At5g24655	*LSU4*	F: CACCATGGGAAAAGGAGGAAACT R: GGGAGAGGCAGAGTCGGAG
At4g35090	*CAT2*	F: CACCATGGATCCTTACAAGTATCGTC R: GATGCTTGGTCTCACGTTCA
At5g65430	*GRF8*	F: CACCATGGCGACGACCTTAAGCA R: TCAGGCCTCATCCATCTGCA
At3g04120	*GAPC1*	F: CACCATGGCTGACAAGAAGATT R: TTAGGCCTTTGACATGTGG
At5g51110	*RAF2*	F: CACCATGGCCGCCACGTCATCAT R: TCACGCCCAAGCTCTTTTCC
At3g22890	*APS1*	F: CACCATGGCTTCAATGGCTGCCGT R: TTACACCGGAACCACTTCTG
At3g49580	*LSU1^C54A^*	F: ACTCGCATCGCAGCTGGCGGAGCTGGR: AGCTCCGCCAGCTGCGATGCGAGTTG
At3g49580	*LSU1^C54E^*	F: GGCGGAAGAGCAACTCGAGTCGCAG R: CTCCGCCAGCTGCGACTCGAGTTG
At3g49580	*LSU1^C54R^*	F: CGGAAGAGCAACTCCGATCGCAG R: CAGCTGCGATCGGAGTTGCTCTT
At3g49580	*LSU1^L60A^*	F: AGCTGGCGGAGGCAGAGGTCGAGT R: ACCTCTGCCTCCGCCAGCTGCGA
–	*GAL4-AD*	F: TACCACTACAATGGATGATGT
–	*GAL4-BD*	F: TCATCGGAAGAGAGTAGTAAC
–	*tADH1R*	R: GAGCGACCTCATGCTATACCT

The series of plasmids for Bimolecular Fluorescent Complementation (BiFC) studies containing LSU1–4 fused N-terminally to N-terminal or C-terminal sections of yellow fluorescent protein (YFP) were generated using pSITE-nEYFP-C1 and pSITE-cEYFP-C1 vectors, respectively (Martin et al., 2009). The expression cassettes for LSU1–4 TAP-tagged at the C-termini were generated using the binary vector pYL436 (Rubio et al., 2005).

### Yeast Two Hybrid (Y2H) Experiments

Y2H analysis was performed in Y2HGold (Takara Bio USA, Inc.) or PJ69-4a (James et al., 1996). *Saccharomyces cerevisiae* strains were transformed with pDEST22 and pDEST32 plasmids containing GAL4 activation (AD) and binding (BD) domains, respectively, according to standard procedures. The yeast were plated and selected on synthetic media lacking leucine and tryptophan (−LT), and protein–protein interactions were tested on media without histidine in the presence of 3-amino-1,2,4-triazole (3-AT) at 3 mM, or 5 mM as indicated (−LTH + 3AT), or without adenine (−LTA). The plates were usually incubated at 30^°^C; however, for longer exposures plates were left on the bench at room temperature (RT).

### Confocal Microscopy

All visual observations were made using a Nikon Eclipse TE2000-E inverted confocal microscope (Nikon Corporation, Japan). Bimolecular Fluorescence Complementation (BiFC), which was used to test protein–protein interactions *in planta*, was monitored three days after agroinfiltration of *Nicotiana benthamiana* leaves with *Agrobacterium tumefaciens* cells (strain GV3101), transformed with combinations of respective plasmids. The plasmids encoded the N-terminal (pSITE-nEYFP-C1) or C-terminal (pSITE-cEYFP-C1) section of YFP linked to the LSUs or other proteins of interest. The 35S::H2B-RFP plasmid (encoding histone 2B fused to red fluorescent protein) was used to visualize the nuclei. *A. tumefaciens* bacteria were grown for 18 h at 28°C in Yeast Extract Broth (YEB) medium supplemented with 10 μg/ml rifampicin and 50 μg/ml spectinomycin (BioShop, Canada) prior to agroinfiltration. Interactions were tested using a 488 nm laser (Sapphire 488-20 CDRH; Coherent Inc., USA) and a 515/30 filter. For RFP, a 543 nm laser (helium–neon laser; Melles Griot, USA) and a 605/75 filter were used. Image data were analyzed using EZ-C1 3.90 FreeViewer (Nikon Corporation, Japan).

### Plant Lines and Growth Conditions

Seeds of *A. thaliana* accession Columbia (Col-0) were obtained from the Nottingham *Arabidopsis* Stock Center (NASC, Nottingham, UK). Transgenic lines were generated by *Agrobacterium*-mediated genetic transformation of Col-0 plants using the floral dip method (Clough and Bent, 1998), with plasmids encoding LSU1-TAP, LSU2-TAP, LSU3-TAP, LSU4-TAP and a fusion-less TAP plasmid, each under the control of the cauliflower mosaic virus 35S promoter. The resultant plant lines were verified by PCR and Western blotting for the presence of the transgene and the associated protein, respectively (not shown).

Seeds were dry sterilized as described previously (Zientara et al., 2009) and stratified at 8°C for 1–2 days before germination. Modified 0.5× Hoagland medium, either full (normal Sulfur, nS) or lacking sulfur (−S) was used for all experiments (Tarnowski et al., 2020). In the −S medium, equimolar MgCl_2_ replaced MgSO_4_. Seedlings were grown on polystyrene Petri dishes, 140 mm in diameter with vents (Thermo Fisher Scientific, USA) on growth medium supplemented by 0.8% agar (UltraPure™ Agarose, Invitrogen). Plates were stored at 22°C, in the photoperiod of 16 h day/8 h night for the number of days indicated below (TAP-MS experiment). The plant material was grown in three biological repetitions for LSU1-TAP and the control TAP line (control II lines) and in two biological repetitions for other lines (LSU2-TAP, LSU3-TAP, LSU4-TAP and the control TAP (control I)).

### Molecular Modeling of LSU Dimer 3D Structures

For each of the four LSU1–4 proteins, coiled-coil regions were predicted using the LOGICOIL server (Vincent et al., 2013), demonstrating that parallel dimers were preferred conformations. For each protein, residues 9–39 were always in a coiled-coil structure, while the contributions of residues 50–63 varied for particular LSUs, in the range 10–15% for LSU1 and LSU3 and up to 50–80% for LSU2 and LSU4. Following predictions, the dimeric structures were modeled for regions covering residues 7–65. The structures of 16 possible LSU1–4 homo/hetero dimers, the topologies of which were adopted from LOGICOIL assignments, were modeled with the aid of the YASARA package (Krieger and Vriend, 2014) using the crystal structure of the coiled-coil domain of *Caenorhabditis elegans* SAS-6 (pdb4gkw) (Qiao et al., 2012). This protein acted as a template for the motif of the two parallel coiled-coils (note that LSU1/LSU4 and LSU4/LSU1 models slightly differed due to the imperfect equivalence of two helices in the template structure). The particular regions of LSU1–4/LSU1–4 pairs were step by step iteratively aligned on the two helices of the template structure, using 51 register shifts for each, with no gaps permitted, which covered approximately half of a super-coil turn of the template structure (see Richter et al., 2016) for the original method application). The resulting 2,601 models were then scored according to the number of leucine side-chains possibly involved in the formation of a leucine zipper motif. Visualization of the method used for molecular modeling of LSU dimers using the LSU1–LSU2 pair as an example is shown in Supplementary Material (**Supplementary File 1**). For each of the 16 complexes (LSU1–4 × LSU1–4), the structures with the highest number of leucine–leucine intermolecular contacts, organized as a leucine zipper, were further tuned by 10 successive rounds of optimization of side-chain rotamers, using FoldX ver 4.0 (Schymkowitz et al., 2005). Finally, the free energy of dimer formation was assessed using FoldX. Structural data of the representative models of the putative 16 LSU–LSU homo- and heterodimers are available in the Supplementary Material (**Supplementary Files 2–19**), in the Protein Data Bank (PDB) format and as the Yasara scene format, which can be interactively viewed using the Yasara viewer program (http://www.yasara.org/viewdl.htm).

### TAP-MS Experiments

Seedlings for TAP-MS experiments were grown as described above for four or 10 days, under either sulfur sufficient (4d_nS and 10d_nS) or sulfur deficient (4d-S and 10d-S) conditions. Approximately, 300 mg fresh plant material was frozen in liquid nitrogen and kept at −80°C until TAP analyses were performed (Rubio et al., 2005). The protocol was scaled down for this procedure (smaller amounts of starting material). Plant protein eluates were analyzed by liquid chromatography (LC) coupled to MS (LC-MS/MS). Precipitated proteins were dissolved in 50 μl 100 mM ammonium bicarbonate buffer, reduced in 0.5 M (5 mM final concentration) tris(2-carboxyethyl)phosphine for 1 h at room temperature (RT), blocked with 200 mM S-Methyl methanethiosulfonate (10 mM final concentration) for 10 min at RT, and digested overnight with 10 ng/ml trypsin (Promega, USA) at 37°C. To stop digestion, trifluoroacetic acid was added at a final concentration of 0.1%. The mixture was centrifuged at 4°C, 14,000 g for 30 min, to remove solids. MS analysis was performed in the Laboratory of Mass Spectrometry, Institute of Biochemistry and Biophysics Polish Academy of Sciences using a nanoAcquity UPLC system (Waters Corporation, USA) coupled to an Orbitrap Elite and QExative MS (Thermo Fisher Scientific, USA). MS was operated in the data-dependent MS2 mode, and data were acquired in the m/z range of 300–2000 units. Peptides were separated on a 180 min linear gradient of 95% solution A (0.1% formic acid in water) to 35% solution B (acetonitrile and 0.1% formic acid). The measurement of each sample was preceded by three washing runs to avoid cross-contamination. The final MS washing run was assessed for cross-contamination between samples. Data were searched with the Max-Quant (Version 1.6.3.4) platform search parameters: match between runs (match time window 0.7 min, alignment time 20 min), enzyme: trypsin/p; specific; max missed 2, minimal peptide length seven amino acids, variable modification: methionine oxidation, N-terminal acetylation, phosphorylation (STY), ubiquitination (GG), fixed: cysteine alkylation, main search peptide tolerance 4.5 ppm and protein FDR 0.01. The reference *A. thaliana* proteome database from UniProt was used (downloaded on 2019.02.04, 39,381 entries). The semi-quantitative analysis of TAP-MS results involved protein intensity comparisons (Orlowska et al., 2013). Protein abundance was defined as the mean signal intensity of a protein calculated by MaxQuant software divided by its molecular weight (Cox and Mann, 2008). Relative specificity was defined as the LOG10 ratio of median protein signal intensity probe to the median intensity of the corresponding signal in the control (the background level was arbitrarily set to one for proteins not detected in the control). The proteins with relative specificity higher than one were defined as the candidates for LSUs molecular partners.

### Other Bioinformatics Tools

Gene annotations were downloaded from the *Arabidopsis* Information Resource (TAIR) and frequencies of functional categorization were calculated using web tool available at the TAIR webpage (https://www.arabidopsis.org/tools/bulk/go/index.jsp). Interaction networks were analyzed with Cytoscape software (Shannon et al., 2003). Gene identifiers were translated using BridgeDb app (Gao et al., 2014).

## Results

### Experimental Evidence for LSU Dimer Formation

**Figure 1:**
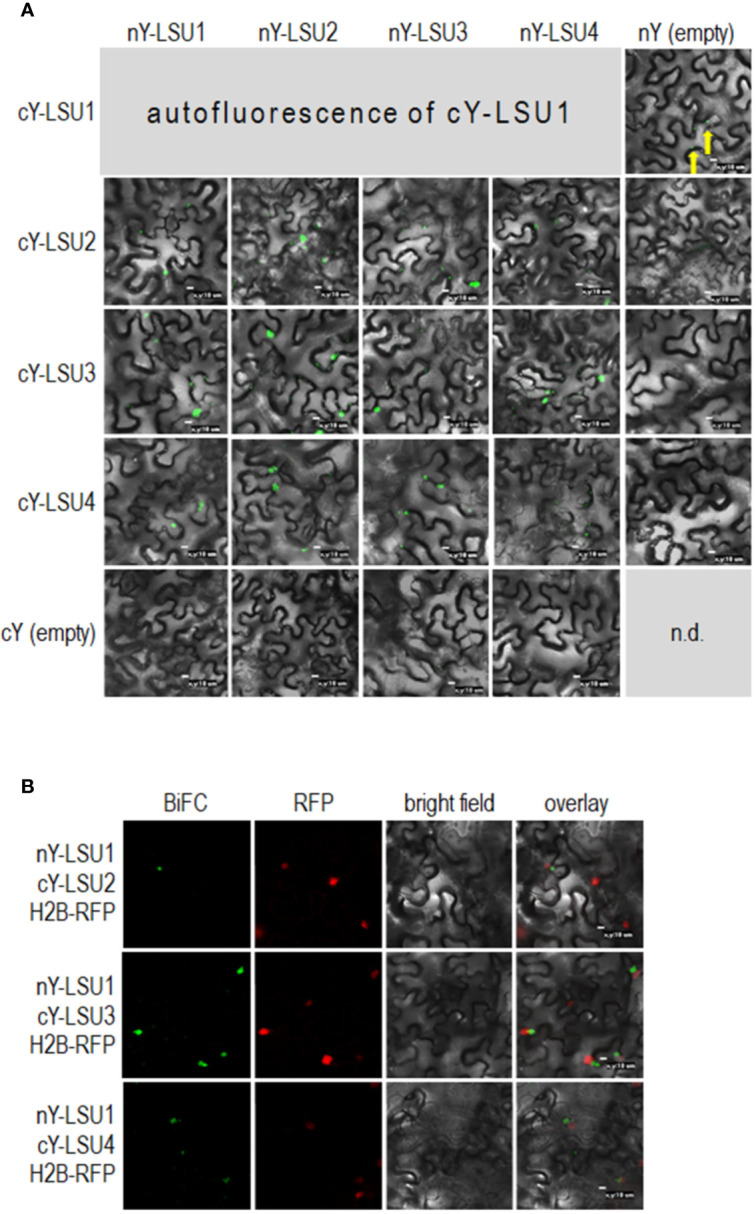
Representative Bimolecular Fluorescence Complementation (BiFC; green spots) image of LSU–LSU pairs **(A)** and co-localization of BiFC for LSU1, with the recombinant nuclear H2B-RFP protein (red spots) **(B)**. cY and nY represent the C-terminal and N-terminal sections of YFP, respectively, fused to the indicated protein or present in empty vector. n.d, no data for this combination. The yellow arrows point to autofluorescence of cY-LSU1. The enlarged versions of the images presented are shown in **Supplementary Figure 1**.

To characterize *A. thaliana* LSU proteins, we investigated their ability to form dimers. Interactions were first examined *in planta* by the BiFC method. Homodimer formation was positively verified for LSU2–LSU2, LSU3–LSU3, and LSU4–LSU4 pairs (**Figure 1A**). Formation of LSU1–LSU1 homodimers could not be tested by BiFC because the YFP signal was already observed in one control combination of LSU1 with the empty vector (CY-LSU1 and NY-pSITE). We also tested the possibility of heterodimer formation by the same method and detected YFP signals for all combinations, suggesting that *in planta* LSU monomers interacted with each other and that all possible mixed interactions may be occurring (**Figure 1A**). We acknowledge that interaction strength based on BiFC observations may be misleading; however, the smaller number and size of spots were observed for most combinations with LSU4 (**Figure 1A**). Essentially, the BiFC data indicated that homo- and heterodimer formation was possible *in planta* by practically all LSU monomers and that LSU1 is prone to non-specific binding (the signal was observed in the negative control). Moreover, two types of spots were observed with different frequencies in all cases, the large aggregates (1–2 per cell) and smaller spots. The large spots were not precisely located in the nucleus, but were in close proximity to this organelle. Examples of H2B-RFP co-localization, used as a nuclear marker, with BiFC of nYFP-LSU1 with cYFP-LSU2, cYFP-LSU3, and cYFP-LSU4 are shown (**Figure 1B**).

**Figure 2:**
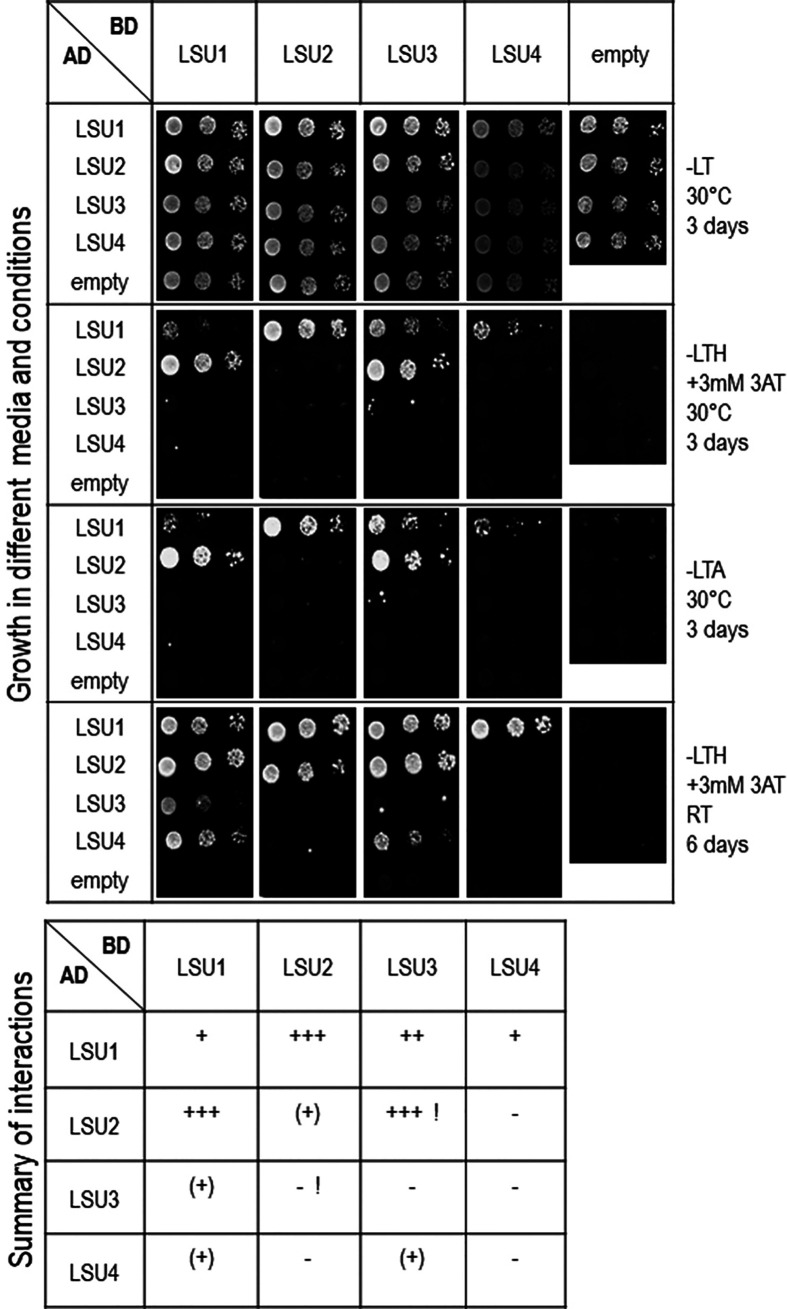
Interactions between LSU–LSU pairs in Y2H experiments. A summary of interactions is shown below the images, illustrating growth of 10-fold serial dilutions of yeast cultures on different selection media. Plates were incubated for three days at 30°C or six days at room temperature (RT), as indicated. The ‘+++’, ‘++’, or ‘+’ reflects the strength of interaction; (+) refers to growth after only six days. Inconsistent results for LSU2 and LSU3 in two combinations of vectors are marked by exclamation marks (!).

To verify LSU–LSU interactions by other methods, we performed a Y2H assay. Using this approach, we observed differences between various homo- and heterodimers (**Figure 2**). Only LSU1–LSU1 and, much less efficiently, LSU2–LSU2 homodimers were determined. Moreover, only LSU1 showed clear interaction with all other LSUs, forming heterodimers in both GAL4 AD and BD fusions where LSU1–LSU2 interactions appeared the strongest. Surprisingly, the LSU2–LSU3 interaction was strong in only one combination of the AD and BD vectors and absent in the reverse combination. Although BiFC showed LSU4 was interacting with other LSUs, the Y2H data suggested it weakly interacted with LSU1 (and LSU3 in one combination).

### Structural Models of LSU Dimers

**Figure 3:**
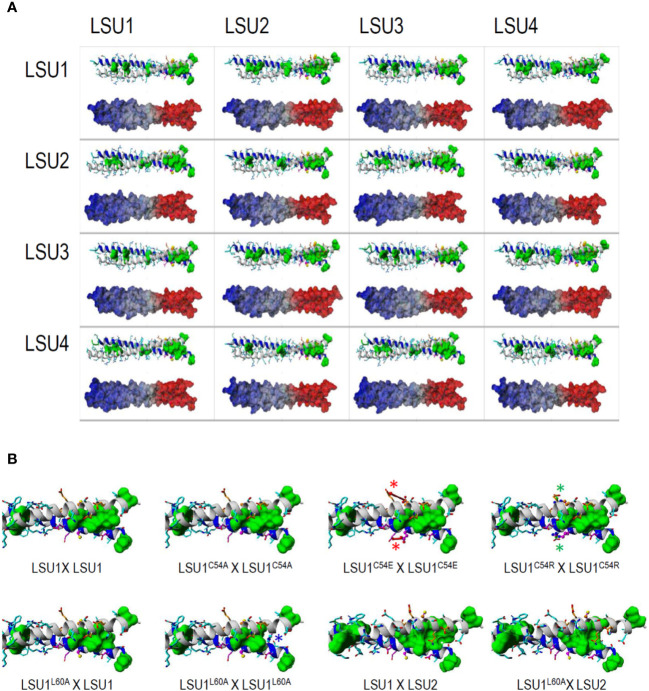
The structures of coiled-coil fragments of the most probable dimers modelled for all 16 possible LSU–LSU pairs **(A)** and the C-terminal part of the coiled coil for the dimeric structures formed by the LSU1 mutants **(B)**. **(A)** According to *in silico* predictions of the coiled-coil regions, protein fragments covering residues 7–65 were modeled. In each inset, the ribbon structure with the side chains—all leucines are marked by green surfaces—is shown above the alternative model demonstrating the distribution of electrostatic potential at the dimer molecular surface; regions of positive and negative potentials are marked in red and blue, respectively. **(B)** C-terminal part of the coiled coil for the dimeric structures of LSU. C54A replacement remains neutral for the coiled coil formation, while the electrostatic interactions of E54/R54 with the proximal E51 destabilize (red arrows and asterisks) or stabilize (green arrows and asterisks) helical structure, respectively, affecting the stability of the dimer. ^L60A^ replacement destroys the leucine-zipper structure in the LSU1^L60A^–LSU1^L60A^ homodimer (blue arrow and asterisk in place of green region), consequently destabilizing the homodimer. In the heterodimers formed by this mutant with LSU1 and LSU2, the leucine-zipper is less affected in LSU1^L60A^–LSU2 than in LSU1^L60A^–LSU1.

The sequence alignments of LSU proteins are shown (**Supplementary Figure 2**). All proteins contained leucine (L) residues, of which seven (L18, L29, L53, L57, L60, L65, and L78) are conserved in all *A. thaliana* LSUs. Additionally, L25 is present in LSU1 and LSU3, L85 in LSU1, LSU2, and LSU3, L37 and L82 in LSU2 and LSU4. Since no 3D structures of LSU-like proteins were available, we built structural models of these proteins. The register shift mapping between the pairs of the 16 variants of LSU dimers indicated that all dimers were possible. However, for the efficient formation of dimers between odd and even LSUs (*e.g.* LSU1–LSU2, LSU1–LSU4, LSU3–LSU2, and LSU3–LSU4, and the equivalent odd-even pairs), the well defined ±4 register relative shift was required (**Supplementary Figure 3**). It means that for odd–odd and even–even LSU dimers, the corresponding residues face each other, while for the odd–even/even–odd LSU dimers the residue “n” of one molecule faces residue "n ± 4" of the second one. Contrary to odd–even/even–odd LSU pairs, the geometry of odd–odd and even–even dimers remained much more flexible. Models of homo- and hetero-dimers of LSU protein fragments spanning the coiled-coil regions (residues 7–63) are shown in **Figure 3A**. Modeling was restricted solely to the predicted coiled-coils regions of LSU proteins. According to these models, the formation of dimers would be possible between all LSU monomers; however, some formations were more preferable than others. Thus, all homodimers and LSU1–LSU3 and LSU2–LSU4 heterodimers were preferably “symmetric”, with no difference in the preferred register shift between the pairs of helices, while the optimal organization of LSU1–LSU2, LSU1–LSU4, LSU2–LSU3, and LSU3–LSU4 heterodimers implied ±4 register shift differences that optimized organization of the leucine zipper (see red thick dotted lines in **Supplementary Figure 3**). Interestingly, the latter complexes were formed by a single combination of register shifts, which reflects structural constraints for these four heterodimers, while homodimers and the remaining four heterodimers could be formed by various combinations of register sifts (*i.e.* 0, ± 4 series) that reflect some extent of conformational heterogeneity. The putative structures of all LSU–LSU dimers, together with electrostatic potentials mapped on the molecular surfaces, clearly demonstrated that particular homo/heterodimers differed minutely in the shape of the coiled-coil region (**Figure 3A**). This also included the spatial distribution of the electrostatic potential, so various dimers may have displayed individual preferences towards particular molecular targets.

### Mutagenesis of LSU1 for Verification of the Predicted Models of LSU-LSU Dimers

All proposed structural models of LSU dimers show that the conserved cysteine (C54) is not involved in dimer stabilization, by the formation of intermolecular S–S bridges. These cysteine residues were not facing each other in any LSU–LSU pair, but they were solvent exposed, and therefore, they (or their particular modifications) may be important for recognizing other target proteins and interacting with the coiled-coil structure. The dimer structures seem to be quite stable; however, the radical changes in this region might have an effect on dimer formation. We designed four changes in LSU1 protein. In three of them, C54 was replaced with alanine (A), glutamic acid (E) and arginine (R), and in the fourth, L60 (an important component of leucine zipper) was replaced with A. The 3D models of the LSU1–LSU1 homodimers with the designed mutations are shown in **Figure 3B**. The model predicted the following effects of the changes on LSU1–LSU1 dimerization relative to the wild type LSU1: C54A—no effect, C54E—destabilization the helical structures and reduced dimer formation, C54R—enhanced dimerization, L60A—reduced dimerization, weaker effect on odd–even/even–odd heterodimers.

**Figure 4:**
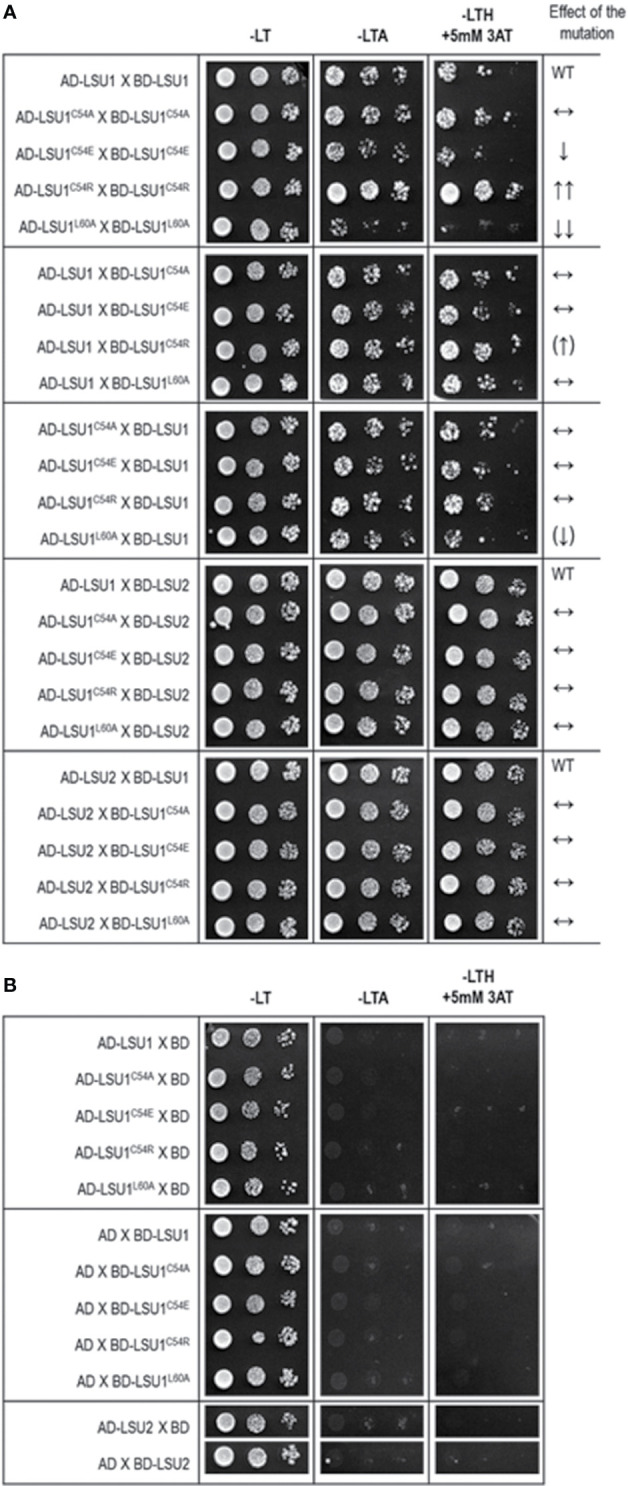
Results of Y2H experiment used to demonstrate the effects of targeted mutagenesis of LSU1 protein on LSU–LSU dimers formation **(A)** and the respective controls **(B)**. The effect of the mutations on dimerization is indicated on the right WT, interaction between the wild type monomers (used as a respective reference), ↔, no effect of the mutation on dimerization; ↓, reduced binding; ↓↓, strongly reduced binding; ↑↑, strongly increased binding; the (↑) and (↓) mark the very weak effects—a tendency towards increase and decrease of binding, respectively.

The accuracy of this prediction was verified in Y2H using the mutated versions of LSU1. Indeed, the Y2H results confirmed the effects of mutations predicted from the molecular models (**Figure 4**). The results were exactly as predicted in the mutant–mutant pairs; however, in the mixed dimers formed by the wild type LSU1 or LSU2 and the mutants, the differences were very weak or unnoticeable, respectively. Nevertheless, experimental data (at least for the homodimers of the LSU1 mutants) were in an agreement with molecular modeling, which additionally supported the proposed models.

### Identification of Proteins Co-Purifying With LSU-TAP

Transgenic *A. thaliana* lines constitutively expressing TAP-tagged proteins (LSU1-TAP, LSU2-TAP, LSU3-TAP, LSU4-TAP, and TAP only; negative control) were used for TAP-MS experiments. The goal of this experiment was to screen for proteins co-purifying with four LSUs in four growth conditions (grown for four days under optimal conditions (4d_nS), or in S-deficient medium (4d-S), or in seedlings grown in the above media for 10 days (10d_nS and 10d-S, respectively)), and to obtain 16 sets of data. The protein extracts were analyzed in either triplicates (LSU1-TAP) or duplicates (remaining LSU-TAP fusions). Analysis of the mass spectrometry proteomics data (deposited at ProteomeXchange Consortium; PXD016023) revealed rather a limited number of candidate proteins (totally 46 in all 16 data sets) co-purifying with LSUs (**Supplementary Table 1**).

**Figure 5:**
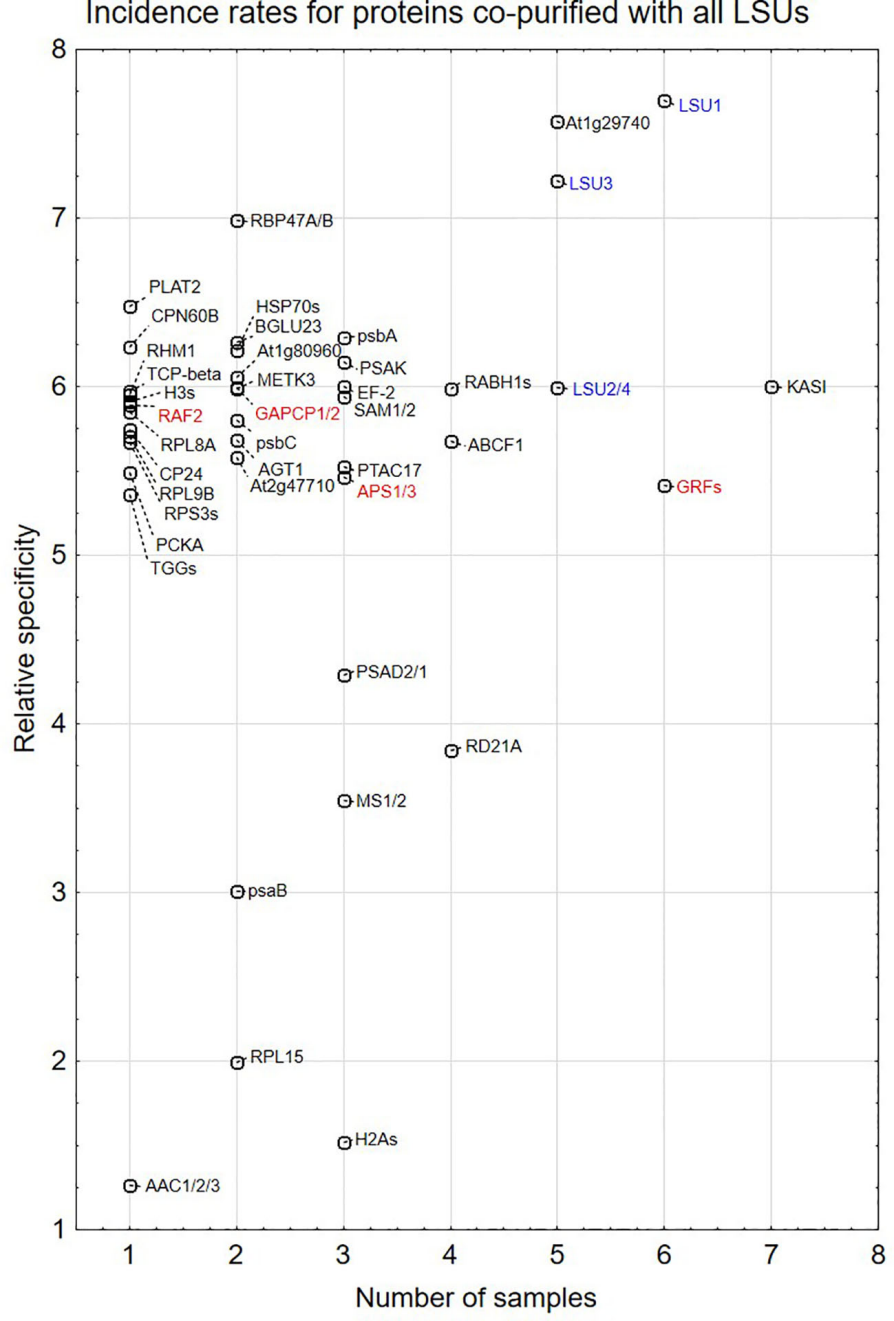
Semi-quantitative plots indicating incidence rates for individual proteins co-purified with all four LSU proteins in four growth conditions (16 biological samples). In some cases, MS results do not distinguish which protein from the family was present in the extract. This is indicated by a “/” or “s” at the end of the name. The proteins are listed in **Supplementary Table 1A**. LSUs detected in the extracts are in blue. Proteins selected for direct interaction verification with LSUs are in red.

**Figure 6:**
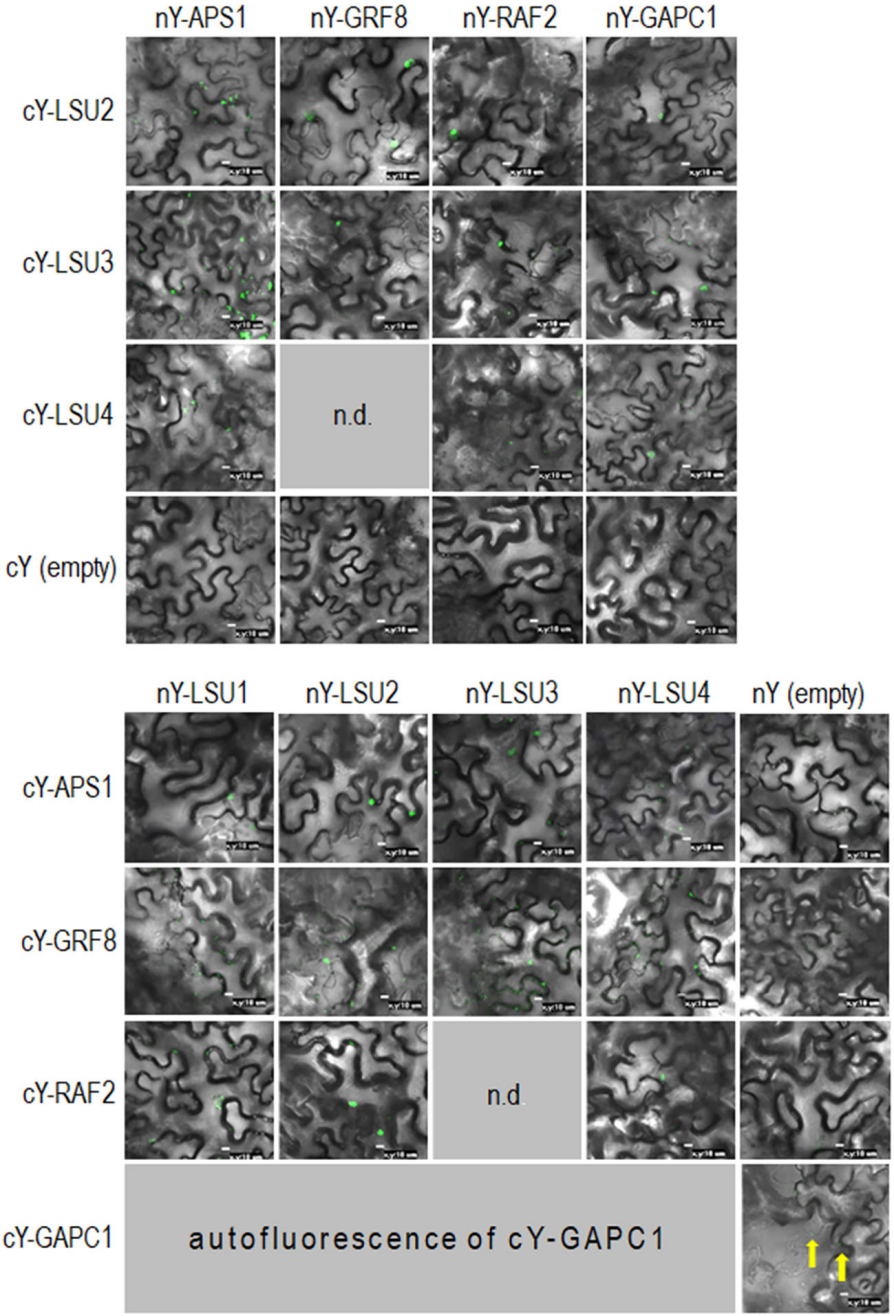
Representative Bimolecular Fluorescence Complementation (BiFC; green spots) of LSUs with APS1, GRF8, RAF2, and GAPC1. The proteins were selected from proteins co-purifying with LSUs in TAP-MS experiments. cY and nY represent the C-terminal and N-terminal sections of YFP, respectively, fused to the indicated protein or present in empty vector. n.d, no data for this combination. The yellow arrows point to autofluorescence of cY-GAPC1. The enlarged versions of the images presented in **Figure 6** are shown in **Supplementary Figure 1**.

**Figure 7:**
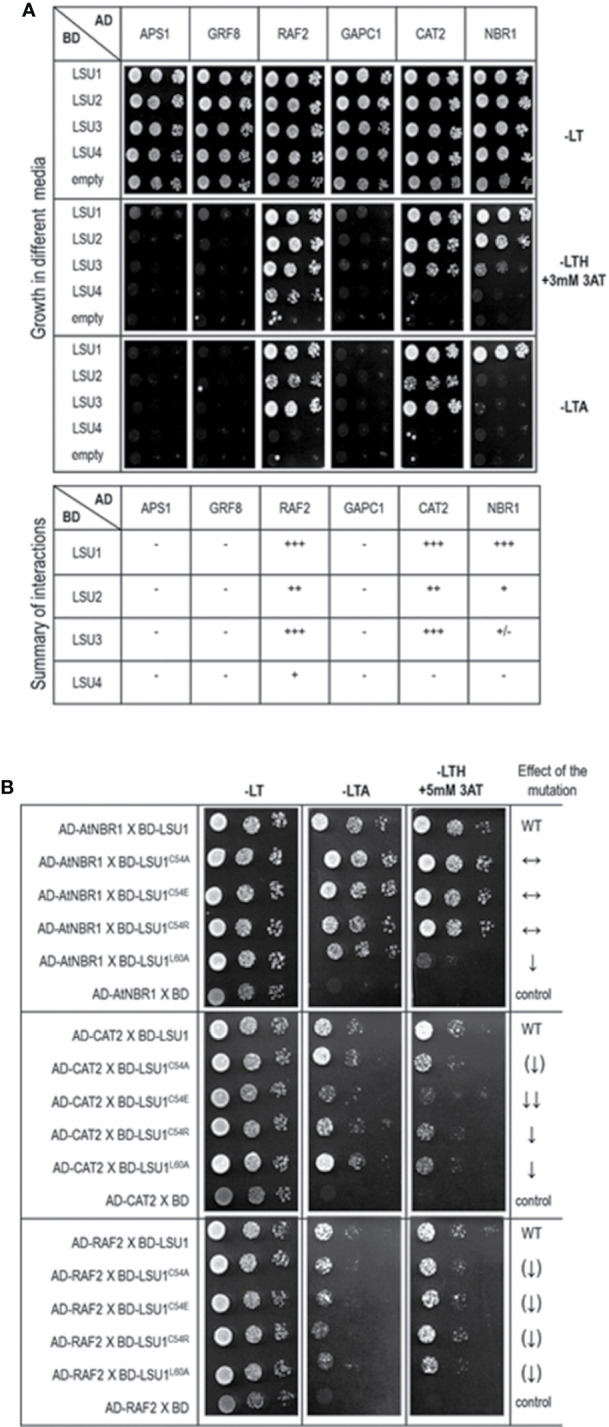
Results of the Y2H experiment used to demonstrate LSU interaction with other proteins **(A)** and effect of the LSU1 mutations on these interaction **(B)**. For **(A)** a summary of interactions is shown below the images, illustrating yeast growth of 10-fold serial dilutions of cultures on different selection media For **(B)** the effect of the mutations on protein interaction is indicated on the right; WT, interaction with wild type LSU1 (used as a respective reference), ↔, no effect of the mutation on interaction; ↓, reduced binding; ↓↓, strongly reduced binding; ↑↑, strongly increased binding; the (↑) and (↓) mark the very weak effects - a tendency towards increase and decrease of binding, respectively.. The plates were incubated for three days at 30°C.

Proteins identified in TAP-MS experiment were analyzed in two ways. First, we checked for the presence of individual LSUs. It is necessary to mention that in contrast to LSU1 and LSU3, the results of MS analysis did not allow distinguishing between LSU2 and LSU4 proteins. Interestingly, the extracts from the LSU1-TAP line contained only LSU1 (the bait) in all tested conditions, in contrast to the extracts from the other LSU-TAP lines, where the respective “bait” was not detected in some conditions, including the absent LSU2 in the LSU2-TAP extracts from 10d_nS and 10d-S, and the absent LSU3 and LSU4 (in the LSU3-TAP and LSU4-TAP extracts, respectively) from 10d-S LSU2 (**Supplementary Table 1A**). These results might indicate that LSU proteins are rather unstable and prone to degradation, especially in the stress (prolong starvation) conditions. Additionally, in some extracts we detected other LSU proteins besides the one used as a “bait”. This result confirms that LSU proteins might form stable heterodimers in plants. Next, we checked for the presence of other proteins in the extract. Most proteins were detected in the extracts from single LSU-TAP lines; however, there were also proteins present in the extracts from more LSU lines. There was no overlap between the set of proteins detected in TAP-MS in this study and the sets of LSU interactors detected previously in Y2H screens. The semi-quantitative analyses of TAP-MS experimental results are shown in **Figure 5**. Because proteins co-purifying with individual LSUs were relatively few, we decided to show the cumulative data for all 16 samples (four LSU baits in four conditions each). This approach seemed to be justified later in the direct interaction tests, which indicated that the proteins identified in TAP-MS experiment as partners of the specific LSU isoform were capable of binding to the other LSUs (see below, **Figures 6** and **7A**). Because of the low number of candidates resulting from the TAP-MS experiment and the failure to detect significant GO term enrichments that could give us any clues for physiological role of LSUs (not shown), our TAP-MS data should be treated rather as a source of potential candidates for LSU interactors to be verified by other methods than as a complete set of proteins co-purifying with LSU.

### Verification of Direct Interactions of LSUs With Selected Proteins

In order to verify if the TAP-MS results revealed the potential partners of LSU, we have picked up four hits from the obtained list of candidates for LSU partners for verification of their direct interactions with LSU1–4 by BiFC and Y2H. The cDNA fragments encoding APS1 (ATP sulfurylase; At3g22890), GRF8 (general regulatory factor 8; At5g65430), RAF2/SDIRIP1/(a protein involved in Rubisco assembly, that also mediates abscisic acid-dependent stress responses; At5g51110) and GAPC1 (C subunit of cytosolic GADPH enzyme involved in the glycolytic pathway, but also possibly involved in a signaling cascade induced by reactive oxygen species; At3g04120) were cloned into the respective vectors. GRF8 was selected as a representative of the 14-3-3 family because it was impossible to distinguish which family members were present in the protein extracts from the TAP-MS experiment. The first test, BiFC experiment, suggested that all proteins may interact with each LSU *in planta* (**Figure 6**). The second test was the Y2H screen. In this experiment, besides the four proteins verified in the BiFC assay, we have also included CAT2 (a peroxisomal catalase; At4g35090) and NBR1 (a selective autophagy cargo receptor; At4g24690). The CAT2 and NBR1 proteins were selected as potential LSU interactors based on our previous unpublished (Niemiro and Sirko) and published (Zientara-Rytter et al., 2011) data, respectively. The Y2H results confirmed direct interactions of three out of six tested proteins with LSUs; namely RAF2/SDIRIP1, CAT2 and NBR1. The interaction strength for each partner and the pattern for LSU1–4 binding were different; however, in all three cases, LSU1 was the strongest binder, while LSU4 was the weakest (**Figure 7A**). The lack of LSU interaction with APS1, GAPC1, and GRF8 in Y2H (despite positive BiFC results *in planta*) suggests that the interaction requires either a plant-specific modification or additional factors mediating (or stabilizing) direct contact of these proteins with LSUs.

Subsequently, we tested the effects of C54A, C54E, C54R, and L60A replacements on LSU1 binding to RAF2/SDIRIP1, CAT2, and NBR1 in Y2H system (**Figure 7B**). Surprisingly, the effects of mutations had different effects on LSU1 interaction with different proteins. For example, binding of LSU1^L60A^ to NBR1 was strongly reduced, while binding of the other mutants to NBR1 was similar to the wild type LSU1. In turn, all mutated version had reduced interaction with CAT2; however, the weakest binder was LSU1^C54E^. Furthermore, the weakest effects of the mutations were observed in the binding tests with RAF2/SDIRIP1. Interestingly, all mutants indicated a slight reduction of binding to this protein. These results suggest that LSUs do not use any particular motifs for binding to different targets and, as suggested by the models, use the shape of coiled coil of the dimers to ensure the specificity of the target’s recognition.

### Network Analysis of the LSU Interactomes

**Figure 8:**
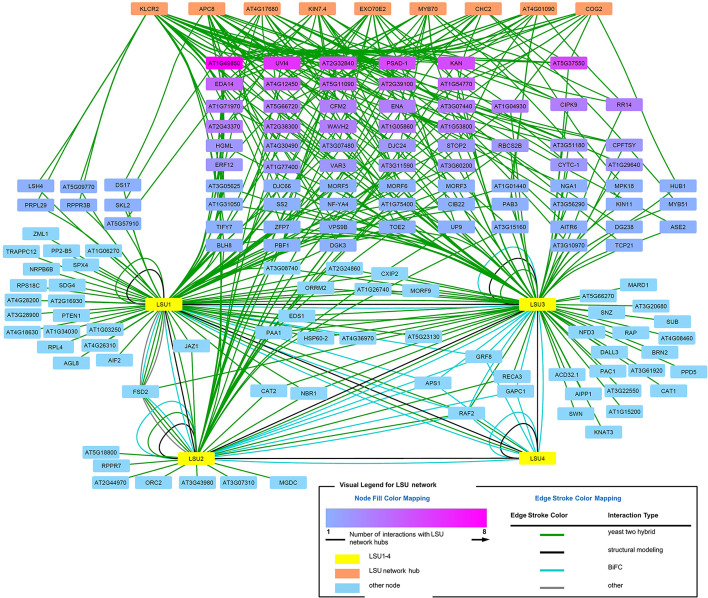
LSU interaction network.

The direct LSU interactors known from the literature (Arabidopsis Interactome Mapping Consortium, 2011; Mukhtar et al., 2011; Garcia-Molina et al., 2017) and those verified in this study by BiFC or Y2H were used for interaction network analysis (visualized in **Figure 8**, see **Supplementary Table 2** for a complete list of interactions**)**. In order to get an insight into the possible function of LSU proteins, we looked for hubs enriched in interaction with the LSUs partners. Using the ratio of the number of LSU interactors to the number of total interactors of the same protein (*i.e.* degree.LSU *vs.* degree.total), we selected the hubs most enriched in interactions with known LSU partners. Using the ratio of the number of LSU interactors to the number of total interactors of the same protein (*i.e.* degree.LSU *vs.* degree.total), we selected nine hubs most enriched in interactions with known LSU partners. The nine selected hubs together with interacting LSU partners are listed in **Supplementary Table 3**. The hubs are ordered according to the number of their interactions with the LSU interactome (LSU.degree). The number of interacting proteins amounts 33, 28, 19, 17, 14, 13, 12, and 10 for the Hubs 1–9, respectively. Hub 1 (KINESIN LIGHT CHAIN-RELATED 2; KLCR2; At3g27960) belongs to the Tetratricopeptide repeat (TPR)-like superfamily and was reported to be involved in pollen tube growth and regulation of defense responses. Hub2 (Anaphase-promoting complex subunit 8, APC8/CDC23; At3g48150) has also the TPR repeat region and is involved in cell division, protein ubiquitination, and regulation of defense response. Hub3 (At4g17680) belongs to the SBP (S-ribonuclease binding protein) family; it has the ubiquitin-protein transferase activity (RING type E3 ligase) and is involved in the regulation of defense response. Hub4 (Kinesin-like protein KIN-7D; KIN7.4; At4g39050) is a microtubule motor protein involved in microtubule-based movement and also in the regulation of defense response and seems to be associated with mitochondria. Hub5 (Exocyst complex component EXO70E2, At5g61010) is involved in defense response by callose deposition, exocytosis, and regulation of protein targeting; it acts as a sequester for cytosolic proteins to release them into the apoplast. Hub6 (MYB family transcription factor MYB70, At2g23290) is involved in the regulation of transcription of not yet identified target genes. Hub7 (Clathrin heavy chain 2; CHC2, AT3G08530) is involved in endocytosis, intracellular protein transport, receptor-mediated endocytosis, and vesicular transport; it is also required for a correct polar distribution of PIN auxin transporters. Hub8 (At4g01090) is a hypothetical protein (with Zn_ribbon domain) reported to participate in wound-induced lateral root development. Hub9 (Conserved oligomeric Golgi complex subunit 2; COG2; At4g24840) is required for normal Golgi morphology and function and is involved in the regulation of exocyst localization and intra-Golgi vesicle-mediated transport. In summary, most of the identified hubs, except those which were not sufficiently characterized, have links to microtubule-dependent transport and regulation of defense response.

We have also noticed that some LSU network proteins interact with more than one newly identified LSU network hubs. When all hub interacting proteins were colored in shades of magenta (**Figure 8**), it turned out that some of them interact with as many as eight hubs (At1g49850) or with six (UVI4). Such dense network of interactions suggests that LSU may interact with protein complexes.

## Discussion

The presented results could be divided into three main groups: (i) Analysis of LSU–LSU dimer formation, molecular modeling, and targeted mutagenesis of LSU1 and dimerization tests of the mutants in Y2H, (ii) Analysis of LSU interaction with other targets, including search for additional candidates for LSU partners by TAP-MS, verification of some candidates by BiFC and Y2H, and testing the effects of targeted mutagenesis of LSU1 on its binding to three targets in Y2H, (iii) Analysis of LSU interactome. This complex approach allowed us to compile the available information necessary to advance our understanding of the role of LSU proteins in plants.

### LSU–LSU Dimers

No information on LSU–LSU oligomerization formations was available so far. We addressed this problem by focusing on the LSU family from *A. thaliana*. Dimer formation was tested by Y2H in *S. cerevisiae* and by BiFC in *N. benthamiana* plants, transiently expressing LSU recombinant proteins. Additionally, molecular models of probable coiled-coiled structures formed by parallel dimers of LSU homo- and heterodimers were also constructed. We failed to correlate the free energy of formation of particular dimers with the Y2H data. The relative changes in the estimated free energy for formation of particular dimers were strongly dependent on the length of fragments used in this analysis, especially on the number of residues flanking the hypothetical coiled-coil region, which differed among structures with alternative register shifts. Generalizing, the interaction of residues flanking coiled-coil regions must contribute significantly to the stability of particular dimers. So, our molecular modeling calculations can be used qualitatively (dimer geometry) rather than quantitatively (free energy of interaction). However, the fact that multiple register shift pairs can form an optimal leucine zipper in all odd–odd and even–even LSU dimers supports the hypothesis that odd–even LSU dimers may be more inert. Bearing in mind that homodimers are preferably formed upon protein synthesis, the formation of odd–even/even–odd LSU heterodimers may be an adjustable platform for a further cascade of interactions with specific targets. The specificity of these interactions is tuned by the combination of the shape and/or dynamics of the coiled-coil structure (*i.e.* difference in register shift between the two helices of the LSU dimers forming coiled-coil) and electrostatic interactions (see the electrostatic potential mapped on the dimer surface; **Figure 3A**).

The experimental and the modeling data in this study allow us to draw the following conclusions: (i) parallel dimers can be formed between different LSU monomers; however, the interaction efficiency differs in different pairs, (ii) LSU dimers are most probably located in the cytosol (not nucleus), (iii) LSU dimers have coiled-coil formations and are stabilized by a leucine zipper, (iv) the conserved cysteine residues (C54) do not form S–S bridge between the monomers of LSU, (v) registry shift is necessary for interactions between “odd” and “even” LSUs, which means the surface of the dimer is affected, and (vi) assuming that LSU–LSU dimers (and not monomers) interact with other proteins, the ratio of individual monomers could influence different types of LSU–LSU dimers, and consequently, they may regulate specificity towards different partners.

In this study we have shown that LSU proteins are able to form hetero- and homodimers. Then, based on the structural models of LSU–LSU dimers, we designed mutations in LSU1 expected to influence dimer formation. The conclusions from the modeling were generally supported by the results of Y2H analysis of the LSU1 mutants. However, in order to understand the mechanistic details of LSU–LSU multimerization, molecular-level experiments are needed.

### LSU Interactions With Other Molecular Targets

Multiple partners for LSU-like proteins were previously identified using either high throughput approaches (Arabidopsis Interactome Mapping Consortium, 2011; Mukhtar et al., 2011; Wessling et al., 2014) or specific focused studies (Moniuszko et al., 2013; Frerigmann et al., 2014; Garcia-Molina et al., 2017). We conducted TAP-MS experiments to identify the candidates for potential partners of different LSU proteins under different growth conditions. We obtained no overlap with LSU1, LSU2, and LSU3 partners identified previously (Arabidopsis Interactome Mapping Consortium, 2011; Mukhtar et al., 2011). There may be several possible reasons for this. Firstly, previously reported partners were identified in different systems (Y2H) when compared to this study (TAP of LSU complexes from plant protein extracts, without prior crosslinking). Secondly, a small amount of starting material used in experiments limited the number of positive hits in the plant extracts. Thirdly, several proteins were detected as co-purifying with only one LSU (incident rate = 1, **Figure 5**); however, such proteins were later shown to directly interact with other LSUs. This suggests that the list of potential 46 partners from the TAP-MS (listed in **Supplementary Table 1A**) is not saturated.

Direct interactions of LSU1–4 with a few candidates were verified by BiFC and Y2H. Essentially, each protein selected for interaction verification was positive by BiFC, while only three proteins were confirmed as direct LSU partners by Y2H. These verified partners were RAF2/SDIRIP1, CAT2, and NBR1. The first was selected from the TAP-MS experiment. The additional partners, CAT2 and NBR1 were included based on our unpublished data (for CAT2) and on our previous report indicating interaction between tobacco homologs of LSU and NBR1 (Zientara-Rytter et al., 2011). Interestingly, the interactions of CAT2 and RAF2/SDIRIP1 with different LSUs were consistently similar. The strongest interactions of CAT2 and RAF2/SDIRIP1 were observed for LSU1 and LSU3, whereas their interaction was much weaker with LSU2 and the weakest (or absent) with LSU4.

Discrepancies between BiFC and Y2H may result from different experimental settings. Yeasts do not express LSU-like proteins; therefore for Y2H experiments only recombinant LSU proteins were expected to be present. For BiFC experiments, the situation was more complex as intrinsic LSU proteins (or rather their tobacco orthologues) were present, which may have interfered with dimer formation from the introduced LSUs. Additionally, it cannot be excluded that LSU proteins form not only dimers, but also higher level multimers. Furthermore, each method used to screen for the interaction (TAP-MS, BiFC, and Y2H) might deal with different amounts of the available proteins, and it could affect the discrepancies between observations in this study. Nevertheless, we could conclude that the interaction of LSU with other molecular targets cannot be linked to any particular motifs in either LSU or the targets. It rather depends on the overall topology and shape of the protein surfaces.

### Additional Clues From Analysis of LSU Interactome

LSU proteins have already been shown to possess large and partially overlapping interactomes (Vandereyken et al., 2018). The physiological implications of LSU interaction partners detected in this study exceed the scope of this work. Partners of LSU proteins, identified here and reported earlier, include proteins involved in different biological processes, in different cellular compartments, and with different molecular functions. However, analysis of the interaction network of LSU interactors allowed identification of significant hubs that, in turn, provided some insight into the possible function of LSUs. Interestingly, among the most significant hubs there are many proteins involved in plant stress response and microtubule-related transport. It would be tempting to speculate that LSU could be responsible for facilitating the intracellular trafficking of different cellular proteins, which indirectly affects plant response to stress. However, direct links of LSU to microtubule-based vesicle transport or to other elements of vesicular transport remain to be investigated.

Considering the extensive interactome of LSU proteins and unclear function of hetero- and homodimers, the physiological role of these stress-related hubs should be analyzed using plants containing single or multiple (triple) *lsu* mutations.

## Data Availability Statement

The datasets generated for this study can be found in the ProteomeXchange Consortium; PXD016023.

## Author Contributions

AN prepared most plasmids and performed experiments with plant material. DC was responsible for MS experiments. JB performed bioinformatics analysis. JP performed molecular modeling. MS performed Y2H assays. AS and AW conceived and supervised the project and designed the study. All authors contributed to the article and approved the submitted version.

## Funding

This work was supported by the National Science Center, Poland, grant No. 2014/15/B/NZ3/04854 and grant No. 2018/31/F/NZ1/02234.

## Conflict of Interest

The authors declare that the research was conducted in the absence of any commercial or financial relationships that could be construed as a potential conflict of interest.

## References

[B1] Arabidopsis Interactome Mapping Consortium (2011). Evidence for network evolution in an Arabidopsis interactome map. Science 333, 601–607. 10.1126/science.1203877 21798944 PMC3170756

[B2] Burkhard P. Stetefeld J. Strelkov S. V. (2001). Coiled coils: a highly versatile protein folding motif. Trends Cell Biol. 11, 82–88. 10.1016/S0962-8924(00)01898-5 11166216

[B3] Clough S. J. Bent A. F. (1998). Floral dip: a simplified method for Agrobacterium-mediated transformation of Arabidopsis thaliana. Plant J. 16, 735–743. 10.1046/j.1365-313x.1998.00343.x 10069079

[B4] Cox J. Mann M. (2008). MaxQuant enables high peptide identification rates, individualized p.p.b.-range mass accuracies and proteome-wide protein quantification. Nat. Biotechnol. 26, 1367–1372. 10.1038/nbt.1511 19029910

[B5] Davletova S. Schlauch K. Coutu J. Mittler R. (2005). The zinc-finger protein Zat12 plays a central role in reactive oxygen and abiotic stress signaling in Arabidopsis. Plant Physiol. 139, 847–856. 10.1104/pp.105.068254 16183833 PMC1256000

[B6] Frerigmann H. Berger B. Gigolashvili T. (2014). bHLH05 is an interaction partner of MYB51 and a novel regulator of glucosinolate biosynthesis in Arabidopsis. Plant Physiol. 166, 349–369. 10.1104/pp.114.240887 25049362 PMC4149720

[B7] Gao J. Zhang C. van Iersel M. Zhang L. Xu D. Schultz N. (2014). BridgeDb app: unifying identifier mapping services for Cytoscape. F1000Res 3, 148. 10.12688/f1000research.4521.1 25110584 PMC4111116

[B8] Garcia-Molina A. Altmann M. Alkofer A. Epple P. M. Dangl J. L. Falter-Braun P. (2017). LSU network hubs integrate abiotic and biotic stress responses via interaction with the superoxide dismutase FSD2. J. Exp. Bot. 68, 1185–1197. 10.1093/jxb/erw498 28207043 PMC5441861

[B9] Hubberten H. M. Klie S. Caldana C. Degenkolbe T. Willmitzer L. Hoefgen R. (2012). Additional role of O-acetylserine as a sulfur status-independent regulator during plant growth. Plant J. 70, 666–677. 10.1111/j.1365-313X.2012.04905.x 22243437

[B10] James P. Halladay J. Craig E. A. (1996). Genomic libraries and a host strain designed for highly efficient two-hybrid selection in yeast. Genetics 144, 1425–1436. 8978031 10.1093/genetics/144.4.1425 PMC1207695

[B11] Krieger E. Vriend G. (2014). YASARA View - molecular graphics for all devices - from smartphones to workstations. Bioinformatics 30, 2981–2982. 10.1093/bioinformatics/btu426 24996895 PMC4184264

[B12] Lewandowska M. Wawrzynska A. Moniuszko G. Lukomska J. Zientara K. Piecho M. (2010). A contribution to identification of novel regulators of plant response to sulfur deficiency: characteristics of a tobacco gene UP9C, its protein product and the effects of UP9C silencing. Mol. Plant 3, 347–360. 10.1093/mp/ssq007 20147370 PMC2845781

[B13] Martin K. Kopperud K. Chakrabarty R. Banerjee R. Brooks R. Goodin M. M. (2009). Transient expression in Nicotiana benthamiana fluorescent marker lines provides enhanced definition of protein localization, movement and interactions in planta. Plant J. 59, 150–162. 10.1111/j.1365-313X.2009.03850.x 19309457

[B14] Maruyama-Nakashita A. Nakamura Y. Tohge T. Saito K. Takahashi H. (2006). Arabidopsis SLIM1 is a central transcriptional regulator of plant sulfur response and metabolism. Plant Cell 18, 3235–3251. 10.1105/tpc.106.046458 17114350 PMC1693955

[B15] Moniuszko G. Skoneczny M. Zientara-Rytter K. Wawrzynska A. Glow D. Cristescu S. M. (2013). Tobacco LSU-like protein couples sulphur-deficiency response with ethylene signalling pathway. J. Exp. Bot. 64, 5173–5182. 10.1093/jxb/ert309 24085579 PMC3830492

[B16] Mukhtar M. S. Carvunis A. R. Dreze M. Epple P. Steinbrenner J. Moore J. (2011). Independently evolved virulence effectors converge onto hubs in a plant immune system network. Science 333, 596–601. 10.1126/science.1203659 21798943 PMC3170753

[B17] Myakushina Y. A. Milyaeva E. L. Romanov G. A. Nikiforova V. Y. (2009). Mutation in LSU4 gene affects flower development in Arabidopsis thaliana. Dokl. Biochem. Biophys. 428, 257–260. 10.1134/S1607672909050093 20848913

[B18] Orlowska K. P. Klosowska K. Szczesny R. J. Cysewski D. Krawczyk P. S. Dziembowski A. (2013). A new strategy for gene targeting and functional proteomics using the DT40 cell line. Nucleic Acids Res. 41, e167. 10.1093/nar/gkt650 23892402 PMC3783193

[B19] Qiao R. Cabral G. Lettman M. M. Dammermann A. Dong G. (2012). SAS-6 coiled-coil structure and interaction with SAS-5 suggest a regulatory mechanism in C. elegans centriole assembly. EMBO J. 31, 4334–4347. 10.1038/emboj.2012.280 23064147 PMC3501224

[B20] Richter M. M. Poznanski J. Zdziarska A. Czarnocki-Cieciura M. Lipinszki Z. Dadlez M. (2016). Network of protein interactions within the Drosophila inner kinetochore. Open Biol. 6, 150238. 10.1098/rsob.150238 26911623 PMC4772809

[B21] Rubio V. Shen Y. Saijo Y. Liu Y. Gusmaroli G. Dinesh-Kumar S. P. (2005). An alternative tandem affinity purification strategy applied to Arabidopsis protein complex isolation. Plant J. 41, 767–778. 10.1111/j.1365-313X.2004.02328.x 15703063

[B22] Ruckle M. E. Burgoon L. D. Lawrence L. A. Sinkler C. A. Larkin R. M. (2012). Plastids are major regulators of light signaling in Arabidopsis. Plant Physiol. 159, 366–390. 10.1104/pp.112.193599 22383539 PMC3375971

[B23] Schymkowitz J. Borg J. Stricher F. Nys R. Rousseau F. Serrano L. (2005). The FoldX web server: an online force field. Nucleic Acids Res. 33, W382–W388. 10.1093/nar/gki387 15980494 PMC1160148

[B24] Shannon P. Markiel A. Ozier O. Baliga N. S. Wang J. T. Ramage D. (2003). Cytoscape: a software environment for integrated models of biomolecular interaction networks. Genome Res. 13, 2498–2504. 10.1101/gr.1239303 14597658 PMC403769

[B25] Sirko A. Wawrzynska A. Rodriguez M. C. Sektas P. (2015). The family of LSU-like proteins. Front. Plant Sci. 5, 774. 10.3389/fpls.2014.00774 25628631 PMC4292543

[B26] Tarnowski L. Collados Rodriguez M. Brzywczy J. Cysewski D. Wawrzynska A. Sirko A. (2020). Overexpression of the Selective Autophagy Cargo Receptor NBR1 Modifies Plant Response to Sulfur Deficit. Cells 9, 669. 10.3390/cells9030669 PMC7140714 32164165

[B27] Usadel B. Blasing O. E. Gibon Y. Retzlaff K. Hohne M. Gunther M. (2008). Global transcript levels respond to small changes of the carbon status during progressive exhaustion of carbohydrates in Arabidopsis rosettes. Plant Physiol. 146, 1834–1861. 10.1104/pp.107.115592 18305208 PMC2287354

[B28] Vandereyken K. Van Leene J. De Coninck B. Cammue B. P. A. (2018). Hub Protein Controversy: Taking a Closer Look at Plant Stress Response Hubs. Front. Plant Sci. 9, 694. 10.3389/fpls.2018.00694 29922309 PMC5996676

[B29] Vincent T. L. Green P. J. Woolfson D. N. (2013). LOGICOIL–multi-state prediction of coiled-coil oligomeric state. Bioinformatics 29, 69–76. 10.1093/bioinformatics/bts648 23129295

[B30] Wessling R. Epple P. Altmann S. He Y. Yang L. Henz S. R. (2014). Convergent targeting of a common host protein-network by pathogen effectors from three kingdoms of life. Cell Host. Microbe 16, 364–375. 10.1016/j.chom.2014.08.004 25211078 PMC4191710

[B31] Zientara K. Wawrzynska A. Lukomska J. Lopez-Moya J. R. Liszewska F. Assuncao A. G. (2009). Activity of the AtMRP3 promoter in transgenic Arabidopsis thaliana and Nicotiana tabacum plants is increased by cadmium, nickel, arsenic, cobalt and lead but not by zinc and iron. J. Biotechnol. 139, 258–263. 10.1016/j.jbiotec.2008.12.001 19111837

[B32] Zientara-Rytter K. Lukomska J. Moniuszko G. Gwozdecki R. Surowiecki P. Lewandowska M. (2011). Identification and functional analysis of Joka2, a tobacco member of the family of selective autophagy cargo receptors. Autophagy 7, 1145–1158. 10.4161/auto.7.10.16617 21670587 PMC3242614

