# Supplementary material for: Similar but Not Identical—Binding Properties of LSU (Response to Low Sulfur) Proteins From Arabidopsis thaliana
Source: Front Plant Sci. 2020 Aug 14;11:1246. doi: 10.3389/fpls.2020.01246 (PMC7456924; doi:10.3389/fpls.2020.01246)
Supplement: Supplementary file 1 [file DataSheet_1.zip › Suppl_Material-R1-v7.pdf]

Supplementary Material

1 Supplementary Data

Supplementary File 1: Visualization of the method (mapping of the coiled-coil registry shift) used for molecular modelling of LSU dimers using the LSU1-LSU2 pair as an example. The method is explained in Materials and Methods (Molecular modelling of LSU dimers 3D structures). All 16 possible dimers were tested for the optimal leucine zipper formation (number of Leu residues involved in coiled coil formation between the tested pairs of parallel LSU monomers). The result of the analysis is shown in Supplementary Figure 2, the structures of the coiled-coil fragments of the representative most probable 16 LSU-LSU homo- and heterodimers are shown in Figure 3, while the structural data for the models are available in supplementary material as pdb files.

Suppl_file1_LSU1-LSU2.mp4

Supplementary Files 2-19: The structural data for the representative models of the coiled-coil fragments of the putative 16 LSU-LSU homo- and heterodimers. The files in the Protein Data Bank (PDB) format and as the Yasara scene format can be interactively viewed using the Yasara viewer program (http://www.yasara.org/viewdl.htm).

Suppl_file2_11_00.pdb,
Suppl_file3_12_u4.pdb,
Suppl_file4_13_00.pdb,
Suppl_file5_14_u4.pdb,
Suppl_file6_21_d4.pdb,
Suppl_file7_22_00.pdb,
Suppl_file8_23_d4.pdb,
Suppl_file9_24_00.pdb,
Suppl_file10_31_00.pdb,
Suppl_file11_32_u4.pdb,
Suppl_file12_33_00.pdb,
Suppl_file13_34_u4.pdb,
Suppl_file14_41_d4.pdb,
Suppl_file15_42_00.pdb,
Suppl_file16_43_d4.pdb,
Suppl_file17_44_00.pdb,
Suppl_file18_16xLSUdimer.sce
Suppl_file19_16xLSUdimer.pdb

2 Supplementary Figures

Supplementary Figure 1. Enlarged versions of images presented in Figure 1 and Figure 6. The images are presented as Suppl. Figure 1a-n below (pages 3-16) The parts of images shown in Fig.1 and Fig.6 are marked by the squares. The scale bars (10 μm) are indicated.

Suppl. Fig. 1a. BiFC images for cY-LSU2 x nY-LSU1, cY-LSU2 x nY-LSU2, cY-LSU2 x nY-LSU3 and cY-LSU2 x nY-LSU4

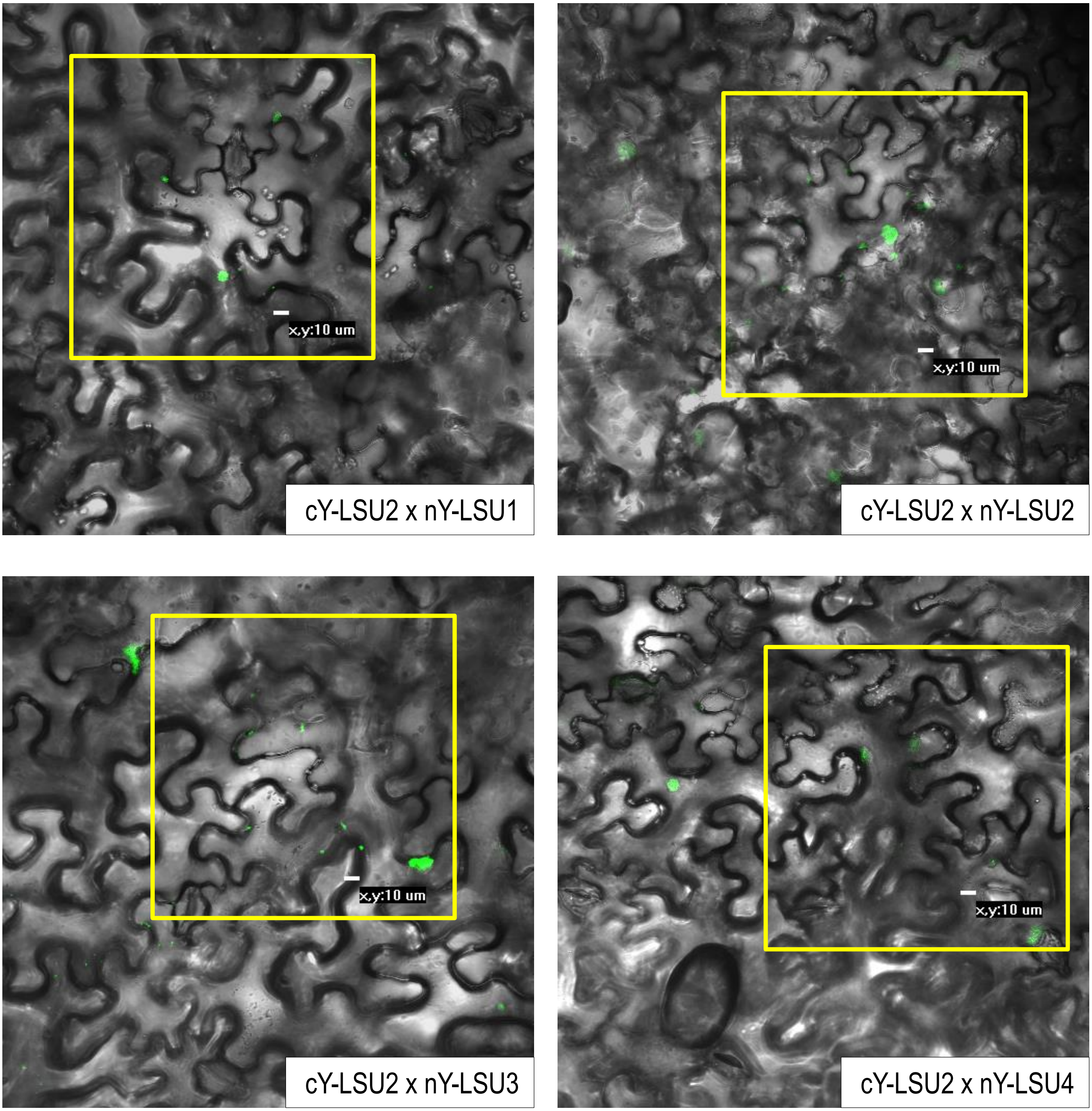

Suppl. Fig. 1b. BiFC images for cY-LSU3 x nY-LSU1, cY-LSU3 x nY-LSU2, cY-LSU3 x nY-LSU3 and cY-LSU3 x nY-LSU4

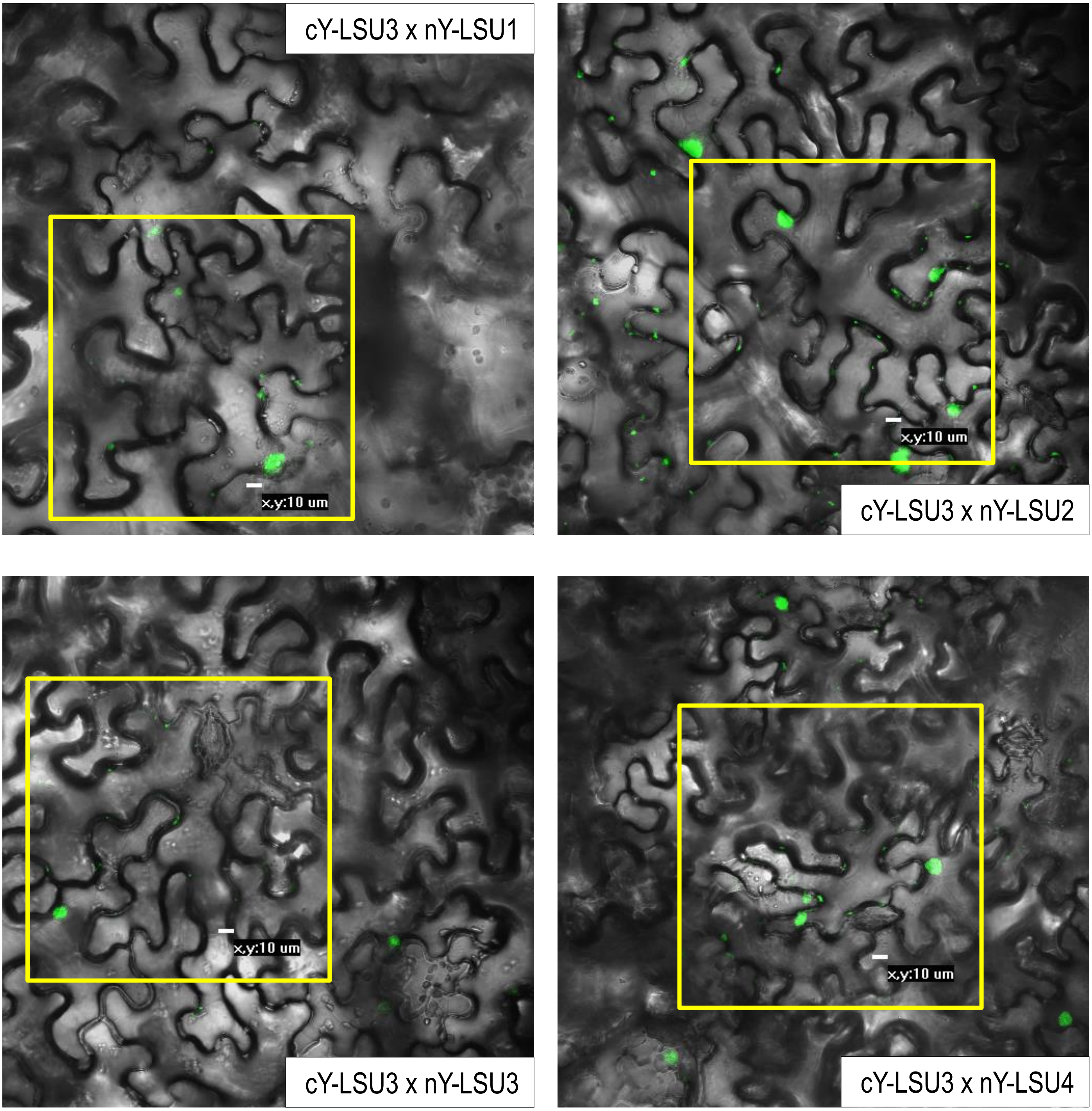

Suppl. Fig. 1c. BiFC images for cY-LSU4 x nY-LSU1, cY-LSU4 x nY-LSU2, cY-LSU4 x nY-LSU3 and cY-LSU4 x nY-LSU4

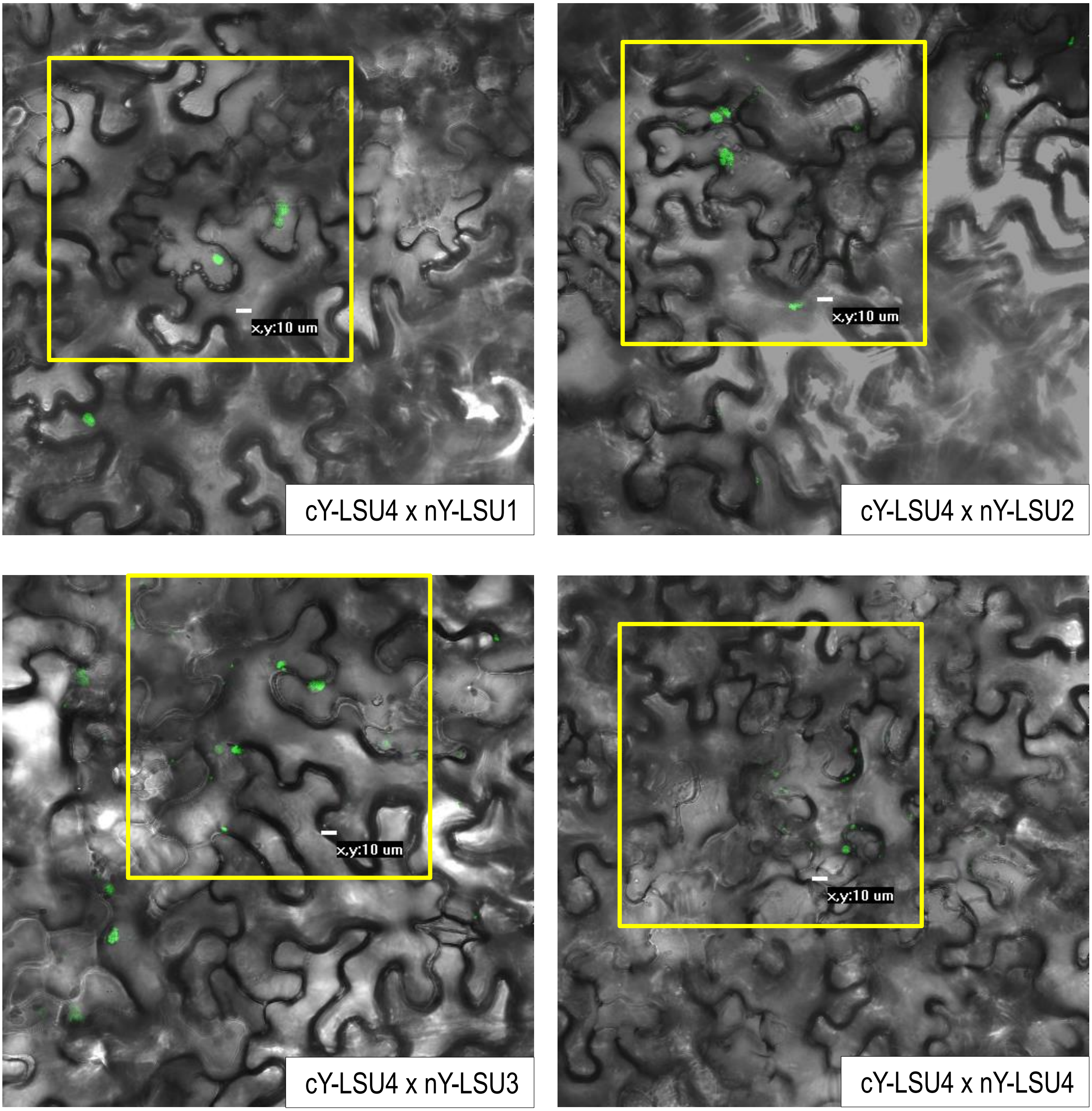

Suppl. Fig. 1d. BiFC images for controls: cY (empty) x nY-LSU1, cY x nY-LSU2, cY x nY-LSU3 and cY x nY-LSU4

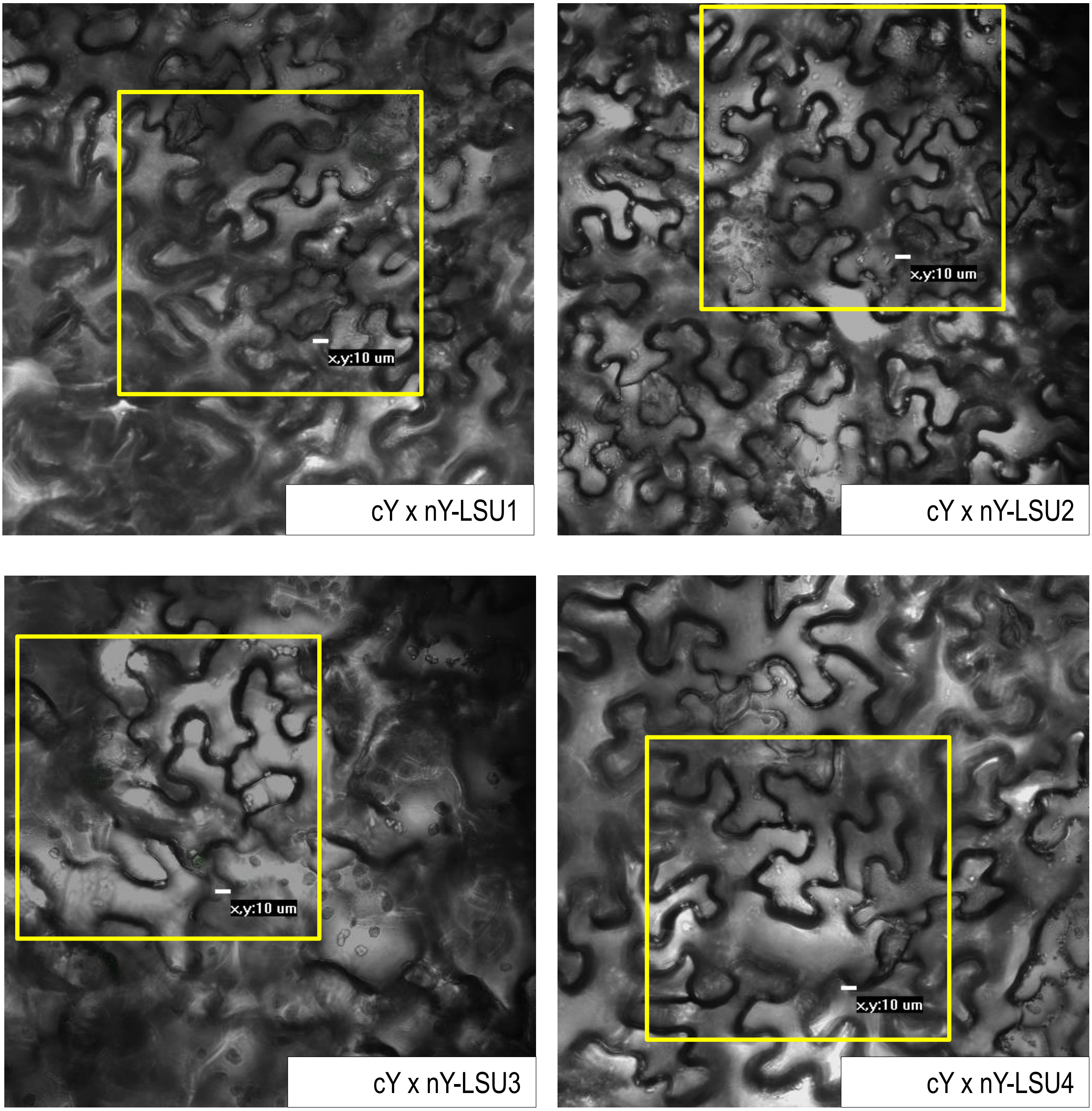

Suppl. Fig. 1e. BiFC images for controls: cY-LSU1 x nY (empty), cY-LSU2 x nY, cY-LSU3 x nY and cY-LSU4 x nY

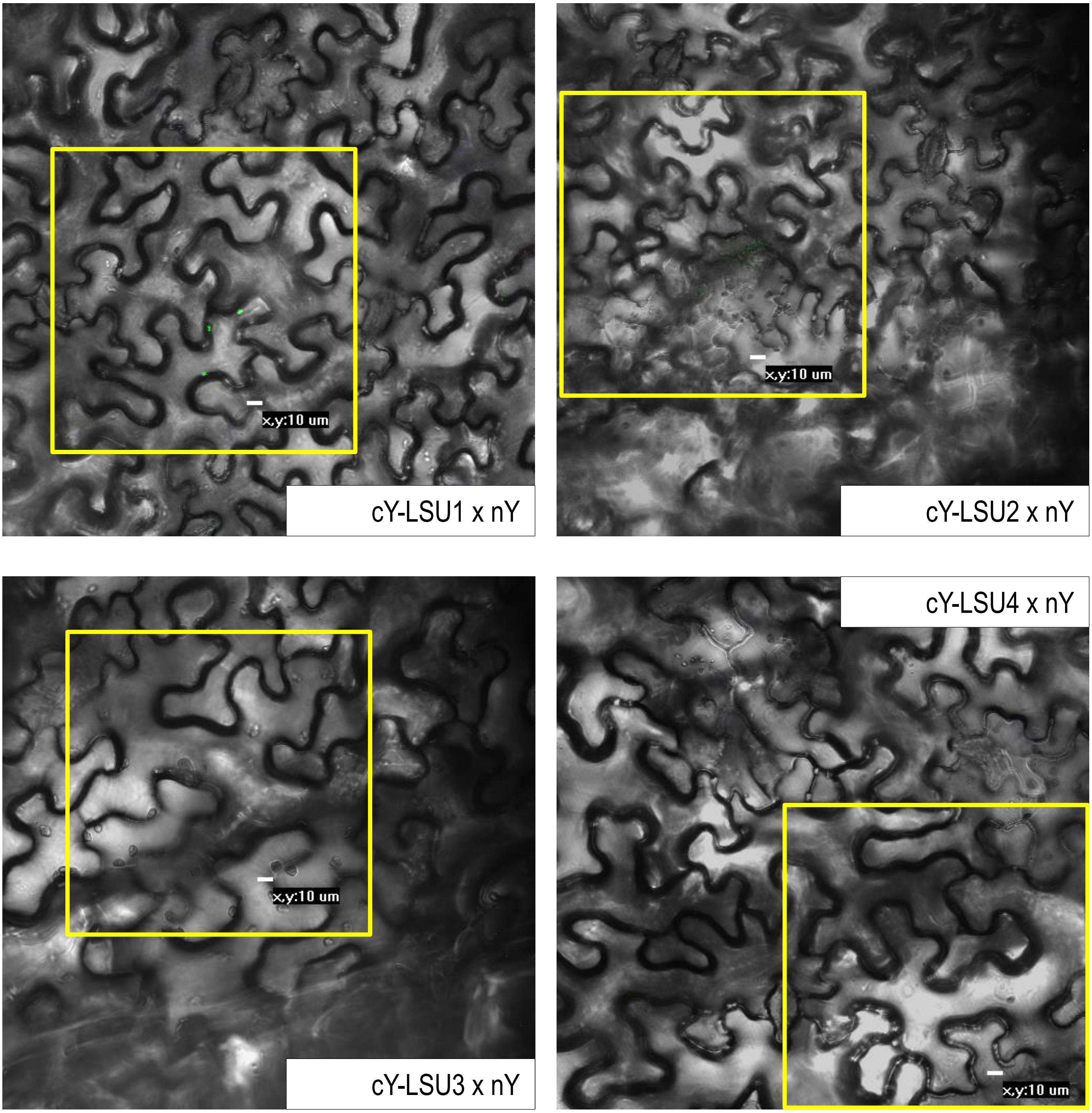

Suppl. Fig. 1f. Representative images of co-localization of BiFC (green spots) for LSU1, with the recombinant nuclear H2B-RFP protein (red spots).

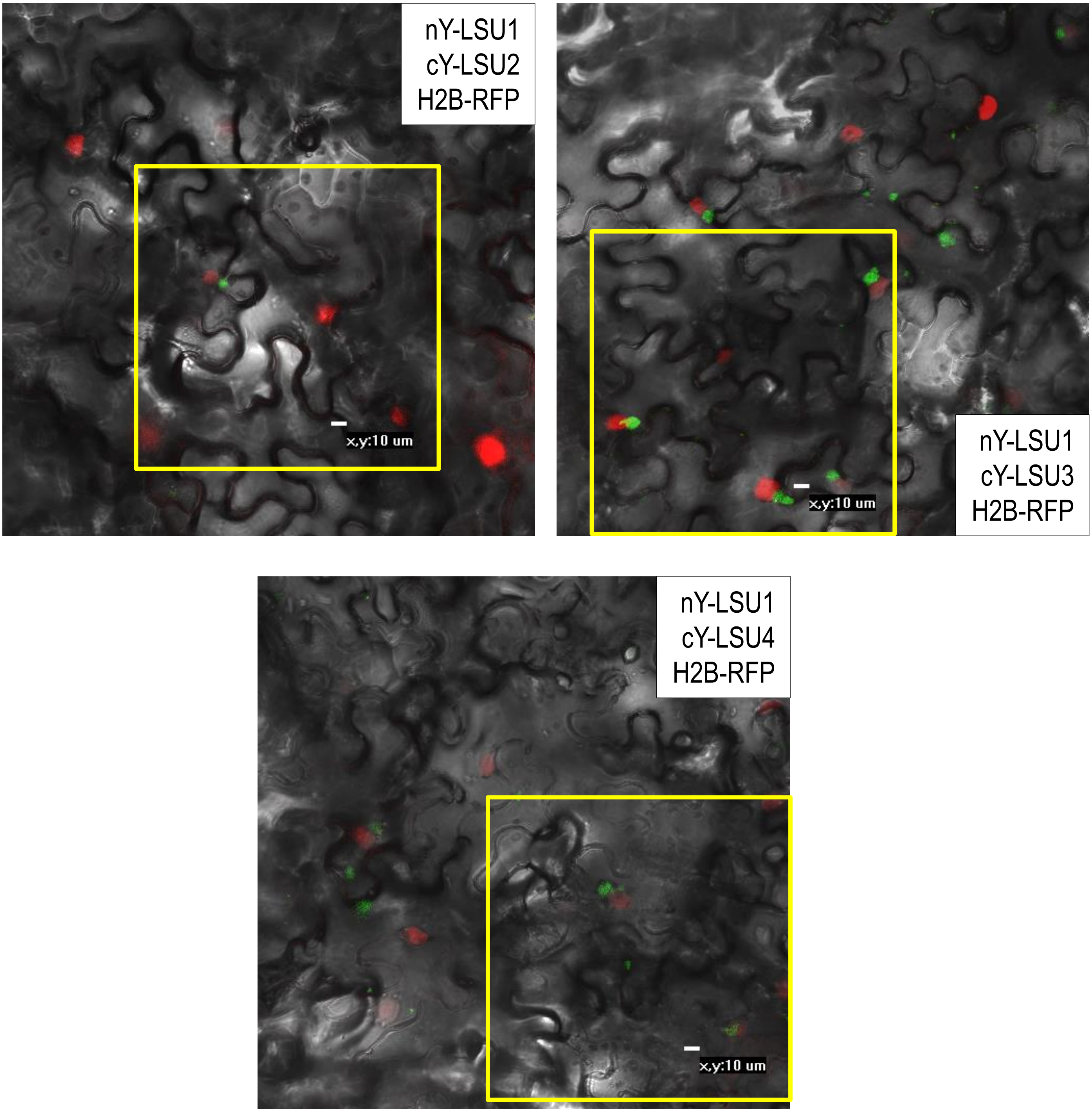

Suppl. Fig. 1g. BiFC images for cY-LSU2 x nY-APS1, cY-LSU2 x nY-GRF8, cY-LSU2 x nY-RAF2 and cY-LSU2 x nY-GAPC1

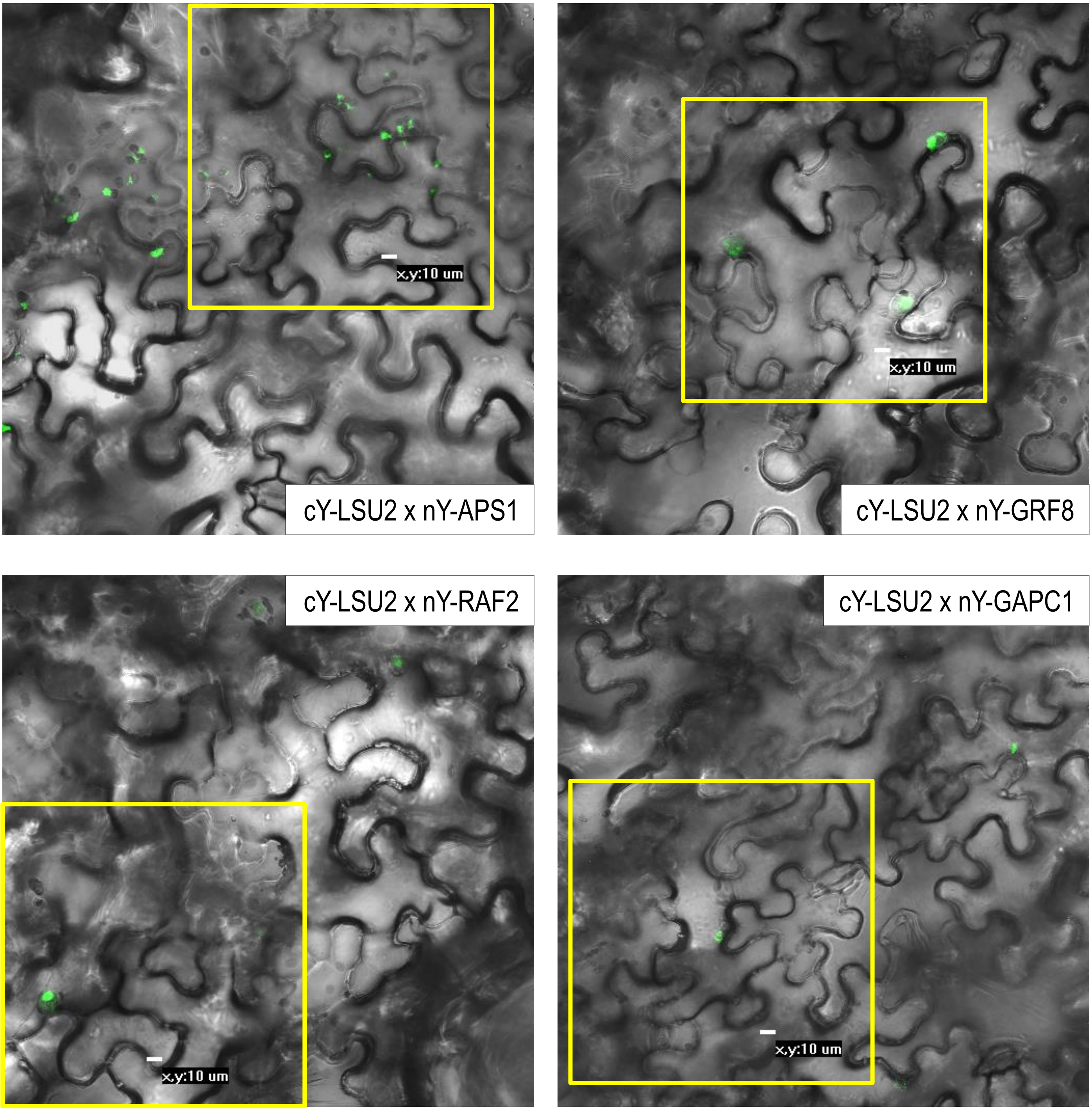

Suppl. Fig. 1h. BiFC images for cY-LSU3 x nY-APS1, cY-LSU3 x nY-GRF8, cY-LSU3 x nY-RAF2 and cY-LSU3 x nY-GAPC1

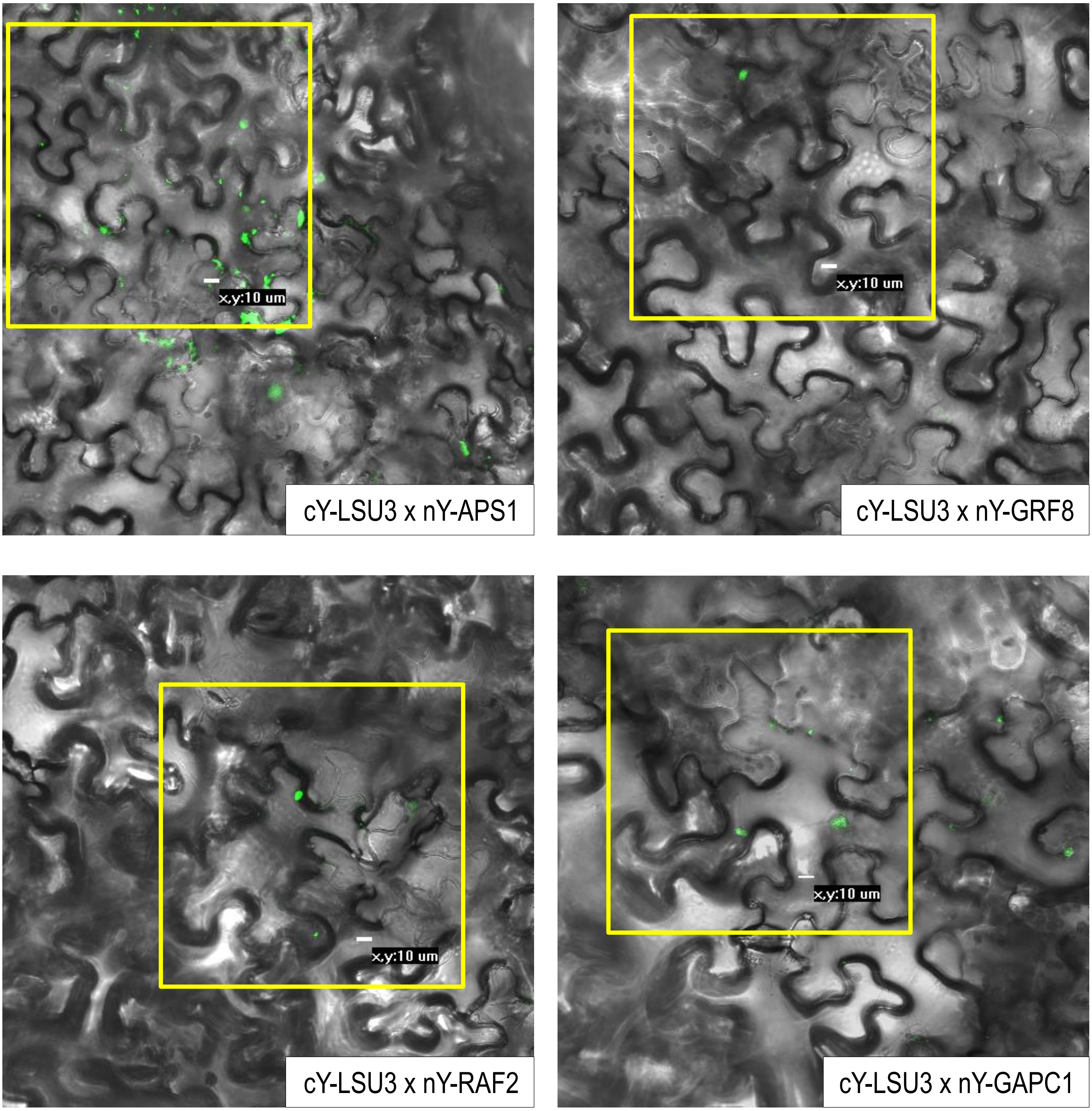

Suppl. Fig. 1i. BiFC images for cY-LSU4 x nY-APS1, cY-LSU4 x nY-RAF2 and cY-LSU4 x nY-GAPC1

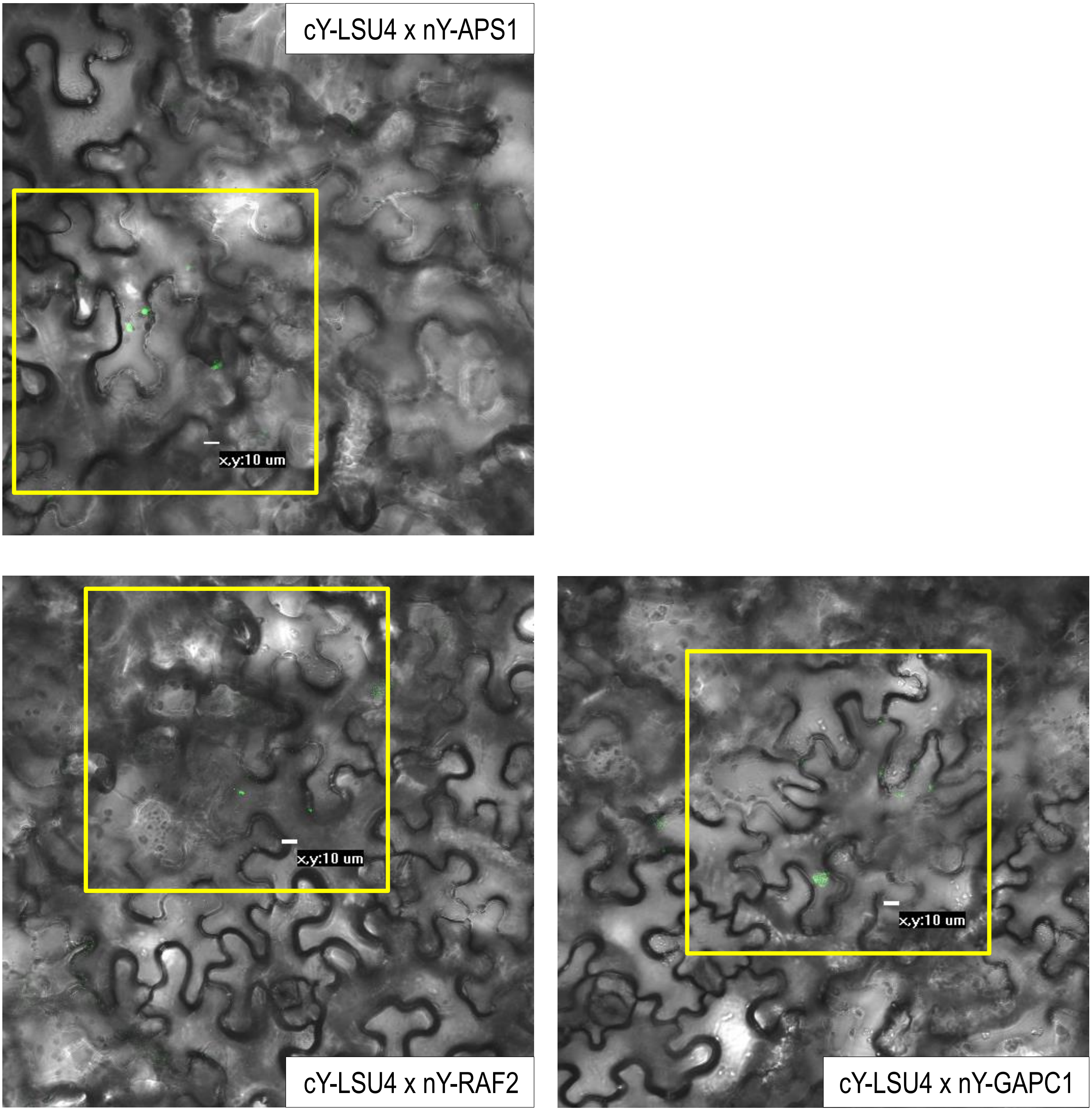

Suppl. Fig. 1j. BiFC images for controls: cY (empty) x nY-APS1, cY x nY-GRF8, cY x nY-RAF2 and cY x nY-GAPC1

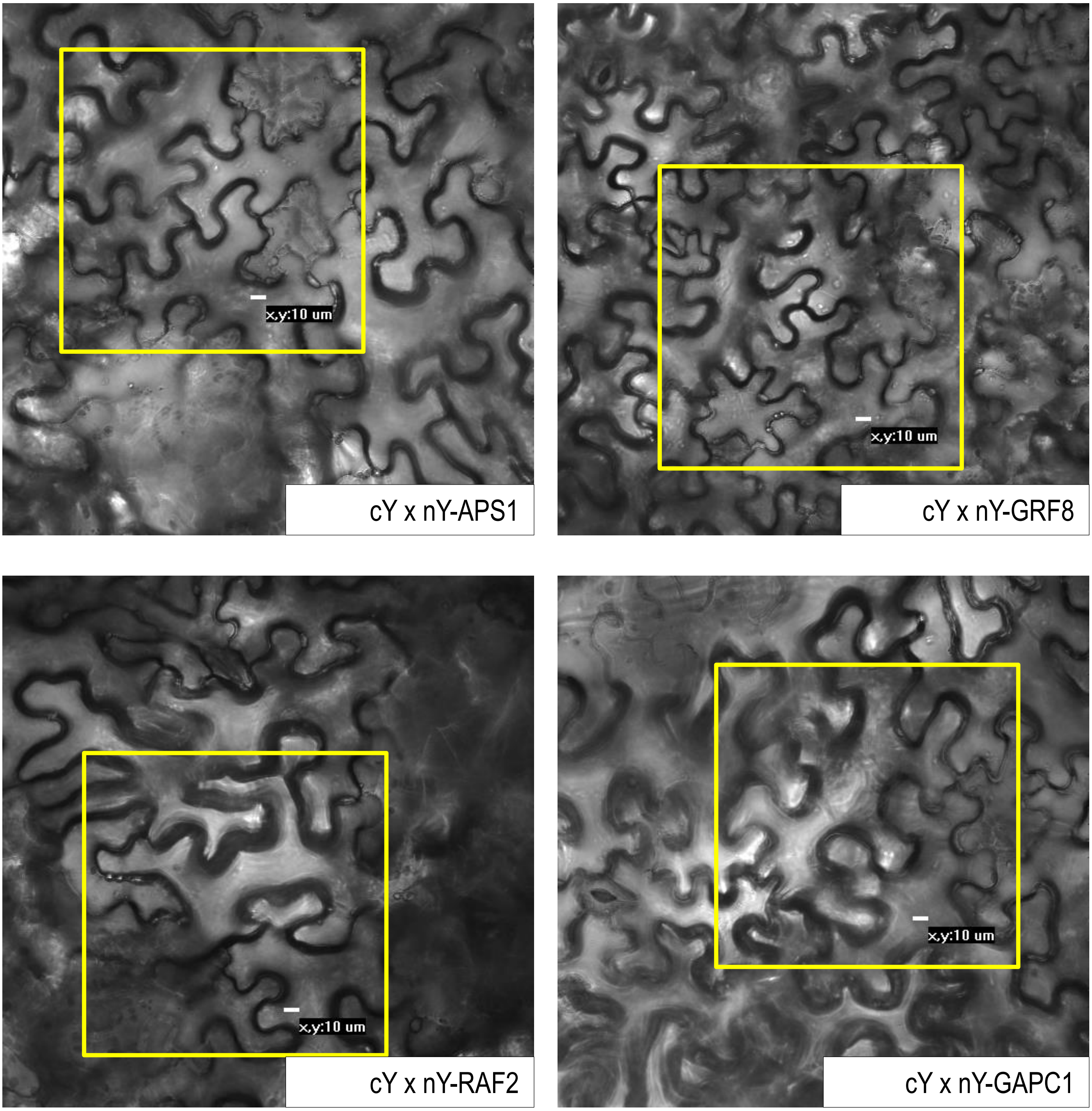

Suppl. Fig. 1k. BiFC images for cY-APS1 x nY-LSU1, cY-APS1 x nY-LSU2, cY-APS1 x nY-LSU3 and cY-APS1 x nY-LSU4

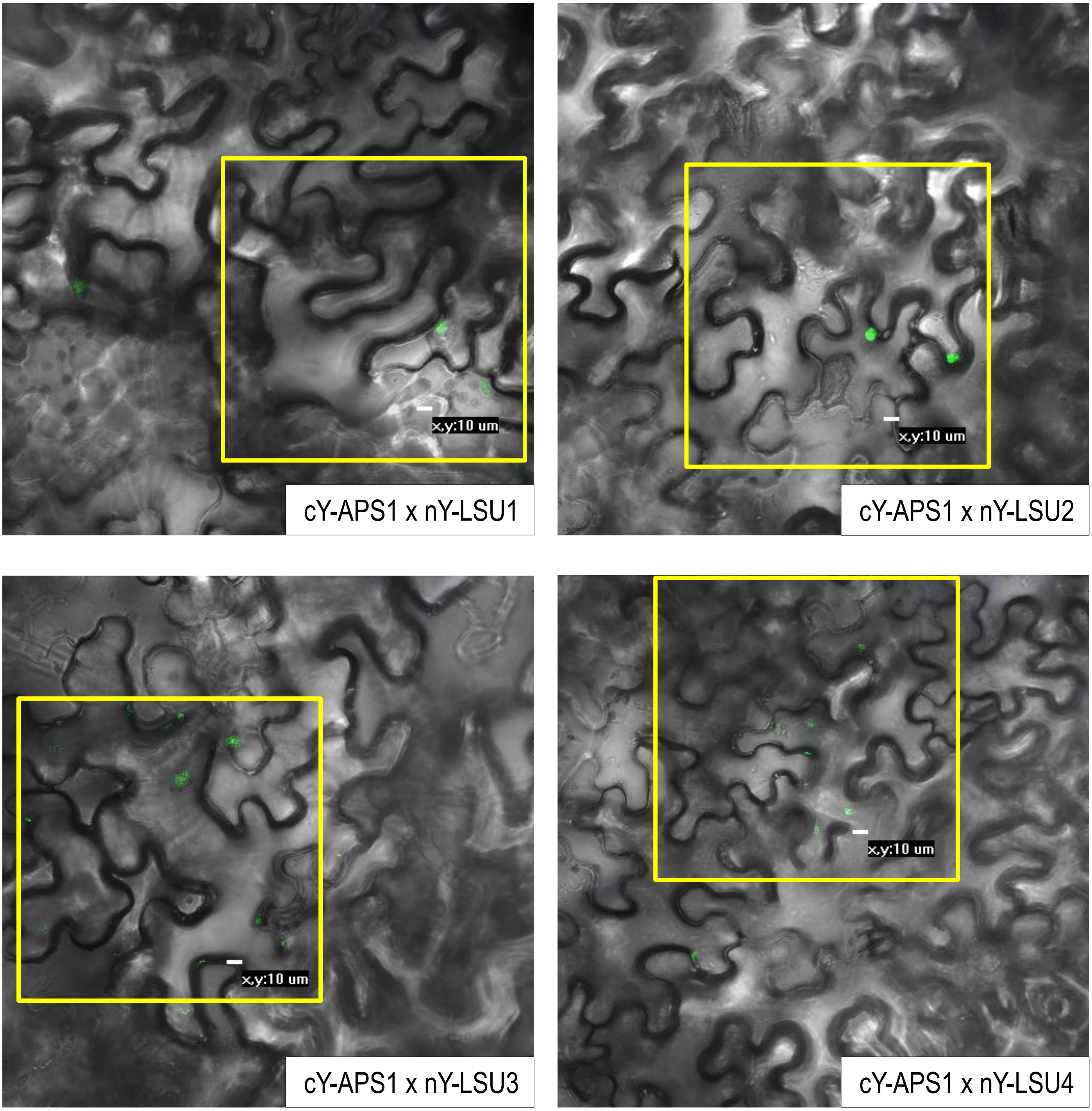

Suppl. Fig. 1l. BiFC images for cY-GRF8 x nY-LSU1, cY-GRF8 x nY-LSU2, cY-GRF8 x nY-LSU3 and cY-GRF8 x nY-LSU4

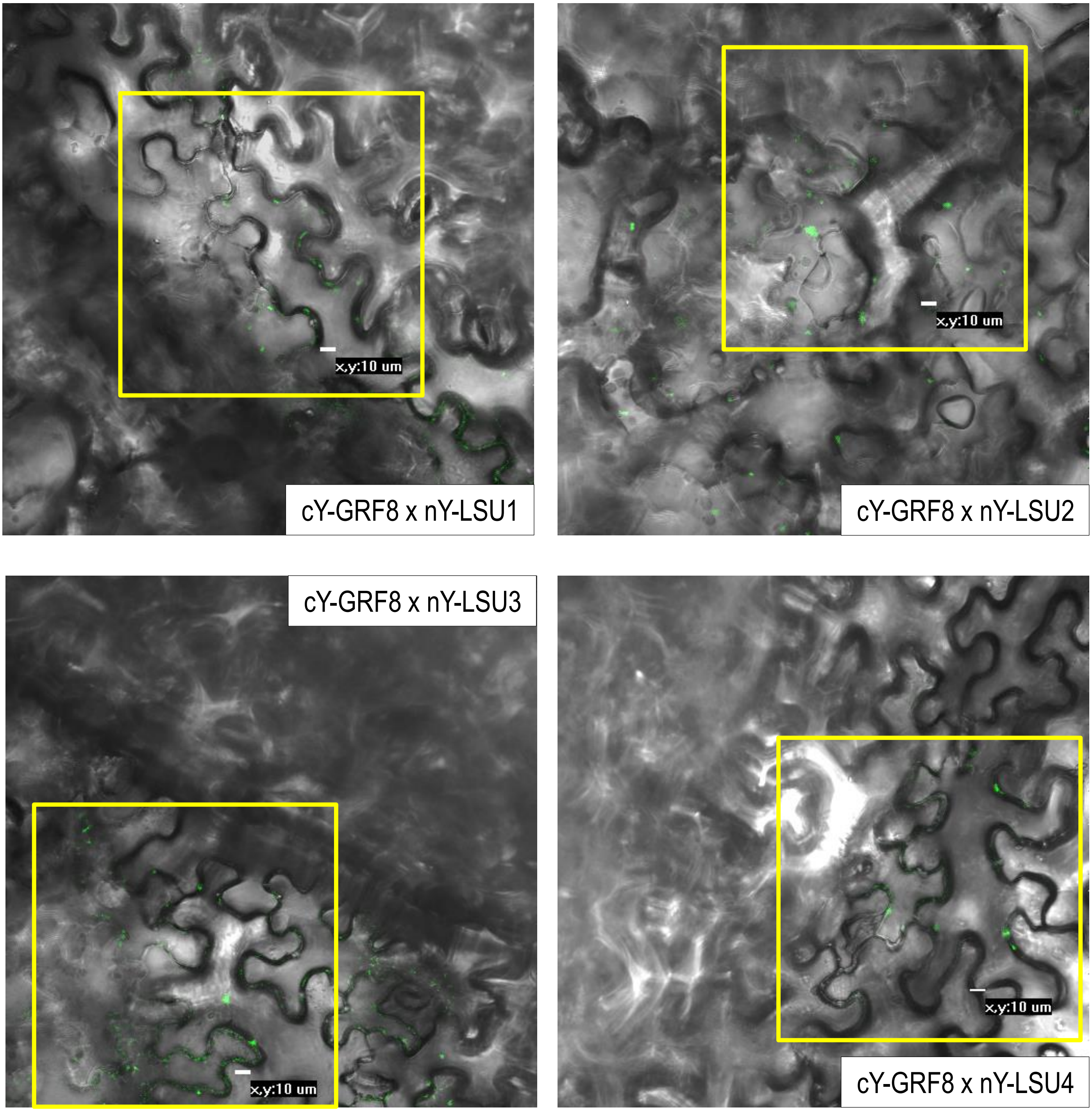

Suppl. Fig. 1m. BiFC images for cY-RAF2 x nY-LSU1, cY-RAF2 x nY-LSU2, cY-RAF2 x nY-LSU3 and cY-RAF2 x nY-LSU4

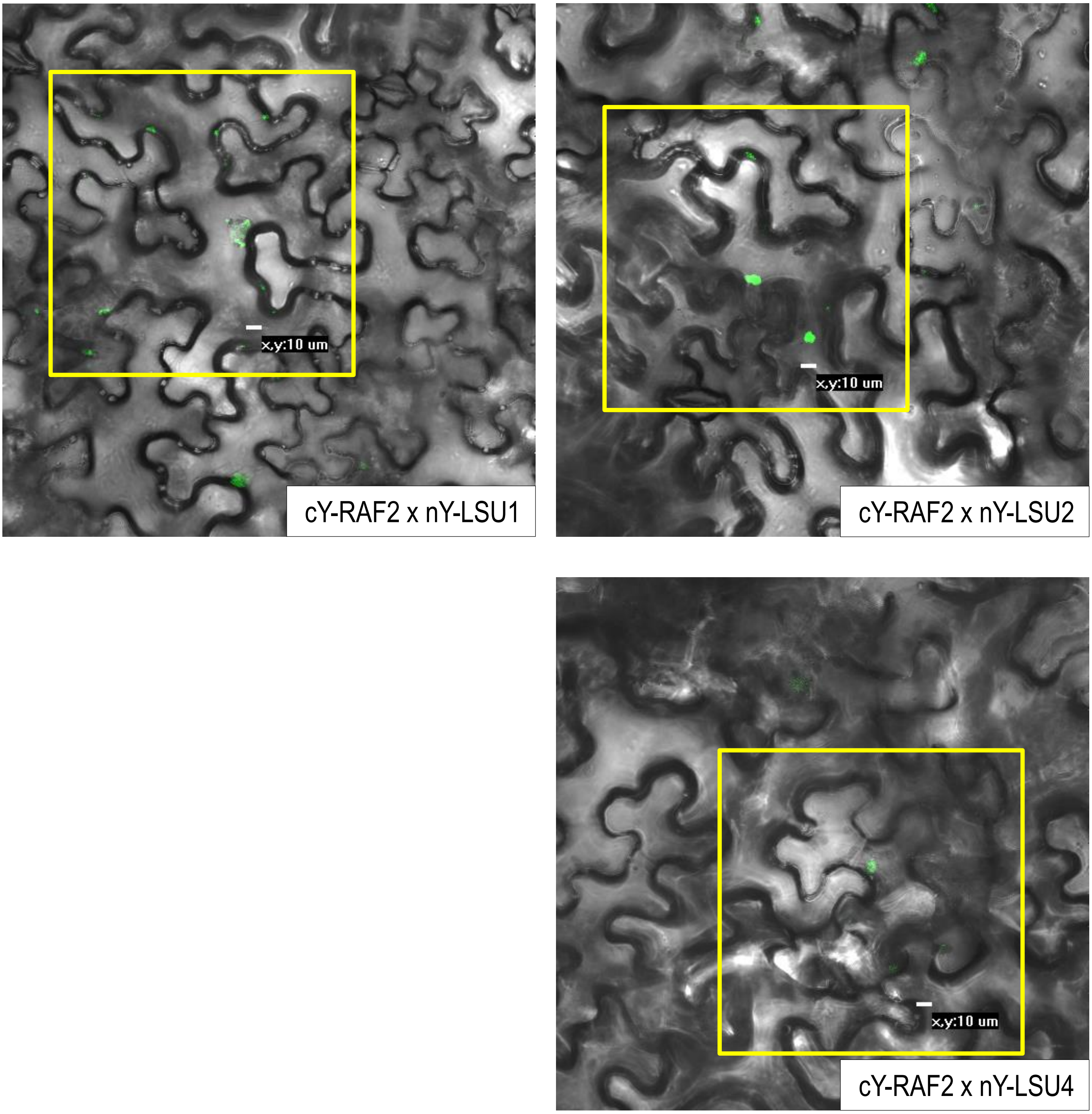

Suppl. Fig. 1n. BiFC images for controls: cY-APS1 x nY (empty), cY-GRF8 x nY, cY-RAF2 x nY and cY-GAPC1 x nY

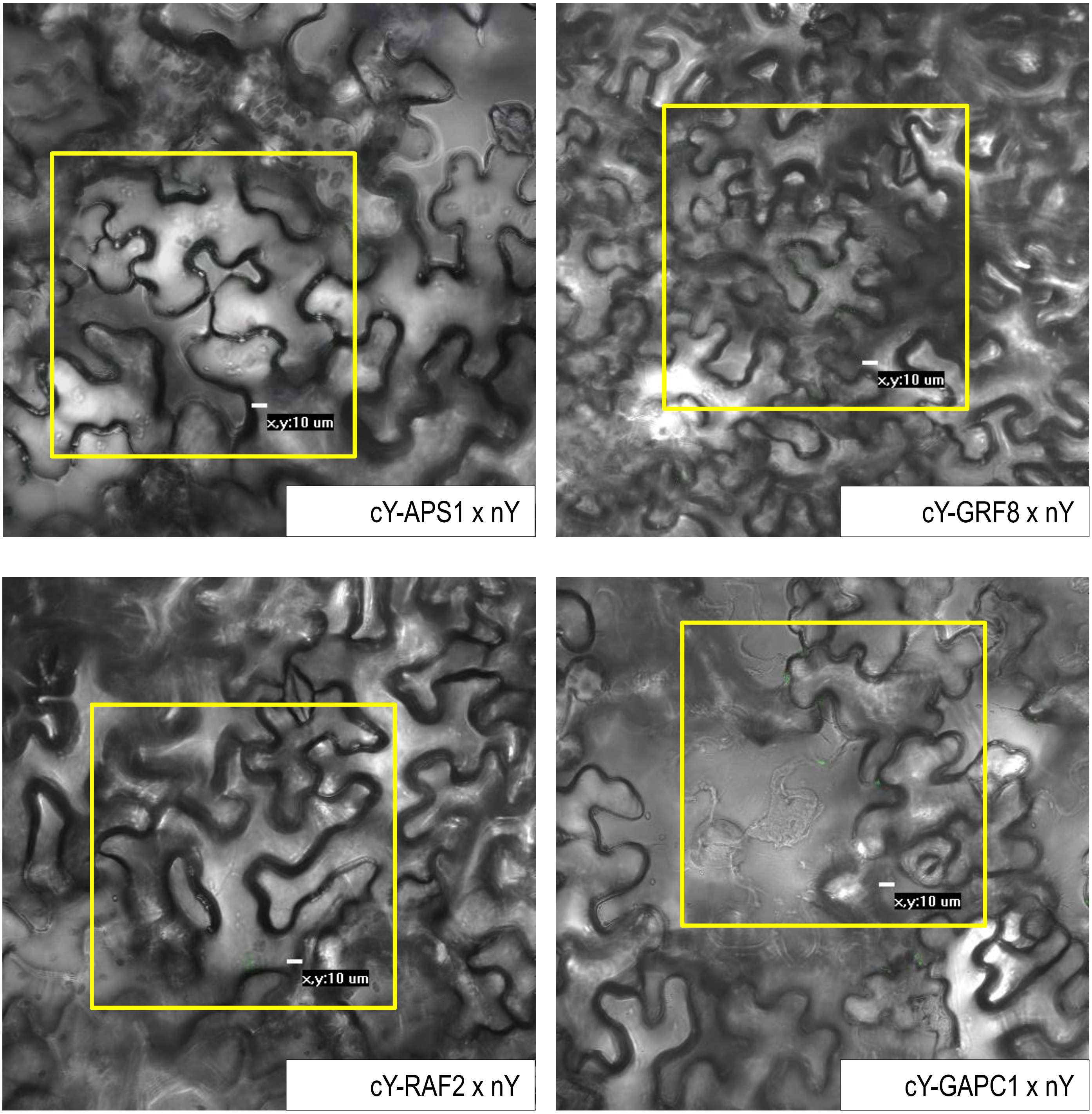

Protein fragments used for molecular modelling of LSU-LSU dimers (residues 7-65)

```
LSU1.2 MANRGGCVTVAAEEMDELRRRNIELSR-------------------EEAEEQLCSQLAELEVESLEQARDYHDRMLFLMDQISRLSSSSVV---SSS
LSU1   MANRGGCVTVAAEEMDELRRRNIELSREVAEMKTEMIKLWQRTVVAEEAEEQLCSQLAELEVESLEQARDYHDRMLFLMDQISRLSSSSVV---SSS
LSU2   MGKGGNYVTVAASEVDELRRKNGEMEKAVEEMKKEMLQLWRRTQVAEEAEERLCSQLAELEAESLDQARDYHSRIIFLMNELSRLSSDSAS---ASP
LSU3   MGKGGGYVTVAAEEVEELRRRNGELEREMEEMKKEMVQLWRRTVVAEEAEERLCSQLAELEVESLDQARDYHSRIVFLMDQISRLSSSSLEVVVTNS
LSU4   MGKGGNYVMVAASEVEELRQKNGEMEKAVEEMRKEMLQLWRRTQVAEEAEEHLCSQLAELEAESLDQARDYHTRIIFLTNQLSRFSSDSAS-----P
cons   *.: *. * ***.*::***::* *:.: : **:.**::**:** *******:*********.***:****** :*:** :::**:**.*       .
```

Supplementary Figure 2. Alignment of amino acid sequences of LSU-like proteins from *A. thaliana* (LSU1-LSU4). Fragments used in molecular modeling are indicated above the alignment. The position of the conserved cysteine (C54) is marked by an arrowhead. Leucine residues involved in zipper formation are marked in green. The red and blue colors indicate positively and negatively charged amino acids, respectively. Two variants of mRNA were reported for LSU1. The first (At3g49580.1) is intron-free, while the second (At3g49580.2) contains one intron and produces a protein with internal deletion covering 19 amino acids (from E28 to A46). The predicted proteins corresponding to At3g49580.1 and At3g49580.2 are shown as LSU1 and LSU1.2, respectively. Only LSU1 was used in the molecular modelling of the dimers.

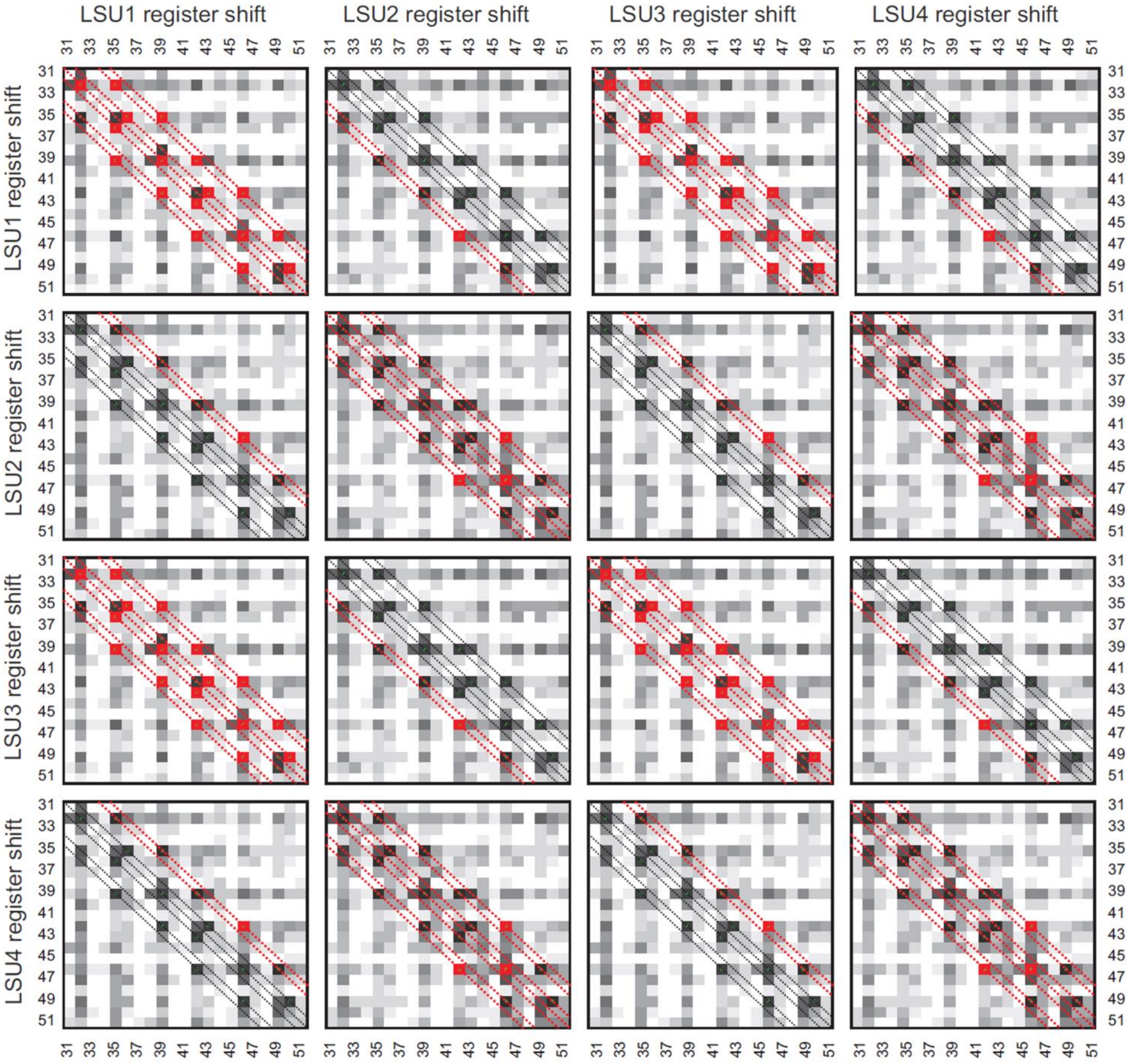

Supplementary Figure 3. Results of register shift mapping between the pairs of helices for all 16 possible LSU-LSU dimers scored by the number of leucine residues involved in the formation of a leucine zipper. Only the most informative part of the maps (~17% of the tested alignments) for LSU proteins are shown. Red squares denote the optimal organization of the leucine zipper, black dotted lines identify the preferred relative in-register shift, and the thick red lines point to the optimal topology of the dimer. The numbers on the scale correspond to the numbers of residues in LSUs.

3 Supplementary Tables

Supplementary Table 1. . Results of TAP-MS experiment. **A.** Proteins selected as specifically detected in the 16 protein extracts. **B**. List of proteins and values describing identification (protein identificator, number of peptides identified for a given protein, sequence coverage, intensity of signal). **C**. List of peptides identified in the experiment. **D**. Parameters search.

Suppl_Table1.xlsx

Supplementary Table 2. List of interactions shown in Figure 8.

Suppl_Table2.xlsx

Supplementary Table 3. LSU Network Hubs (proteins sharing interactomes with the LSU proteins). The LSU interacting proteins having also known interactions with each Hub are listed below the Hub name and description.

Suppl_Table3.xlsx
